# Atlastin-2-mediated endoplasmic reticulum membrane tethering is critical for flavivirus replication

**DOI:** 10.1371/journal.pbio.3003556

**Published:** 2026-09-15

**Authors:** Jonathan Einterz Owen, Cheyanne Lynn Bemis, Qingyi Wang, Ambarish C. Varadan, Jacob W. Vander Velden, Laura Andačić, Olus Uyar, Mansi Gupta, Christopher D. Scharer, Laurent Chatel-Chaix, Pietro Scaturro, Mehul S. Suthar, Christopher J. Neufeldt

**Affiliations:** 1 Department of Microbiology and Immunology, School of Medicine, Emory University, Atlanta, Georgia, United States of America; 2 Center for Childhood Infections and Vaccines, Children’s Healthcare of Atlanta, Division of Infectious Diseases, Department of Pediatrics, Emory University School of Medicine, Atlanta, Georgia, United States of America; 3 Emory Vaccine Center, Emory University, Atlanta, Georgia, United States of America; 4 Leibniz Institute of Virology, Hamburg, Germany; 5 Centre Armand-Frappier Santé Biotechnologie, Institut National de la Recherche Scientifique, Laval, Quebec, Canada; 6 German Center for Infection Research, Hamburg-Borstel-Lübeck-Riems, Hamburg, Germany; 7 Centre for Structural Systems Biology (CSSB), Hamburg, Germany; 8 Faculty of Mathematics, Informatics and Natural Sciences, Department of Biology, University of Hamburg, Hamburg, Germany; University of Oxford, UNITED KINGDOM OF GREAT BRITAIN AND NORTHERN IRELAND

## Abstract

Flaviviruses (genus *Orthoflavivirus*) are arthropod-borne viruses which cause approximately 400 million annual global infections in humans. Flavivirus infection requires cellular machinery to facilitate replication and spread. All known flaviviruses replicate in association with the host endoplasmic reticulum (ER), where genome replication is confined within virus-induced ER invaginations called viral replication organelles (vROs). Despite the central role of these structures during flavivirus infection, the mechanisms underlying vRO biogenesis remain undefined—particularly the membrane rearrangements required for their formation. In this work, we report a conserved role for a cellular ER remodeling protein, atlastin-2 (ATL2), in the organization of vROs within infected cells. Using confocal and electron microscopy, we show that ATL2 depletion leads to a reduction in vRO spatial distribution in flavivirus-infected cells. Changes in vRO distribution corresponded with a decrease in virus production and robust induction of innate immune responses. We also demonstrate that ATL2 accumulates in areas of vRO formation during flavivirus infection. Critically, mutational analysis showed that a tethering-competent but fusion-defective ATL2 mutant was sufficient to rescue DENV and ZIKV replication in ATL2-knockout cells. Finally, targeting of ATL2 activity using synthetic peptides significantly reduced DENV replication in both immortalized and human primary cells, suggesting a possible avenue for targeting host ER functions to limit flavivirus replication. Taken together, these results show that membrane tethering plays a critical and conserved role in flavivirus infection, functioning to organize membranes for vRO biogenesis and limit cellular immune activation. Importantly, we provide evidence that ATL2-mediated membrane organization can be targeted to inhibit viral replication.

## Abstract 2

Flaviviruses reshape cell membranes to build replication sites, but how these membranes are organized remains unclear. This study shows that homotypic membrane tethering driven by the host endoplasmic reticulum protein atlastin-2 is essential for organizing these sites and supporting viral replication.

## Introduction

*Orthoflavivirus* (formerly *Flavivirus*) is a genus of arboviruses that contains numerous pathogens of significant consequence to human health, including West Nile virus (WNV), Zika virus (ZIKV), and dengue virus (DENV). These pathogens are responsible for an estimated 400 million human infections per year, with more than 80% of countries and half the world’s population at risk for infection [1]. The global prevalence of flaviviruses, combined with their high mutation rates, makes recurrent epidemics or novel emergence events likely [2,3]. This is exemplified by the recent emergence, or re-emergence, of flaviviruses such as WNV, yellow fever virus (YFV), Powassan virus (POWV), and ZIKV into new human populations, highlighting the potential for novel flaviviruses to become highly dangerous to human hosts [3–8].

Given their relevance to human health and lack of commercially available antiviral therapies, there is a pressing demand for therapeutic interventions that treat both emerging and endemic flaviviruses [9]. Host-targeted antivirals, which disrupt conserved virus-host interactions, offer an attractive possibility for broad-spectrum drug development—particularly because the barriers to resistance are theoretically higher than those of direct-acting antivirals [10–12]. Therefore, identifying host pathways that are critical for a wide array of virus infections represents an important avenue for antiviral intervention.

Replication of the flavivirus positive-sense single-stranded RNA genome occurs in association with the endoplasmic reticulum (ER) of host cells. More specifically, flavivirus genome replication is presumed to occur inside spherical virus-induced alterations of the ER membrane called viral replication organelles (vROs) [13–15]. The formation of vROs is highly conserved during flavivirus infections, with remarkable similarity in membrane morphology observed across disparate flaviviruses [16]. A vRO consists of a membranous vesicle formed by invagination of the ER into its luminal space, with a diameter of 70–90 nm and a connection to the cytosol through a pore-like opening [14,17–20]. Compartmentalization of viral genome replication within vROs is thought to serve several purposes, including concentrating the necessary proteins and substrates for RNA replication, coordinating trafficking of newly synthesized viral RNA, and shielding viral replication intermediates from immune receptors [17,21–27]. Morphological data and comparisons with other positive-sense single-stranded RNA viruses suggest that new copies of the viral genomic RNA exit the vRO through the pore-like opening and are then engaged in one of two distinct viral processes: translation by ribosomes to produce viral proteins and form new vROs, or packaging into assembling viral particles [28–33]. However, the molecular mechanisms of flavivirus vRO biogenesis and the regulation of viral RNA trafficking in infected cells remain unclear.

De novo vRO biogenesis and virion assembly typically occur on ER membranes proximal to sites of existing vROs [15,18,19,27]. The close connection between ER membranes during viral processes is thought to ensure efficient RNA movement while minimizing exposure of the viral genome to cytosolic RNA sensors and the RNA degradation machinery of the innate immune system [24,34,35]. However, the mechanisms that facilitate these membrane contacts and how this membrane organization specifically supports infection remain undefined. In previous work, we and others implicated an ER-resident membrane remodeling protein called atlastin-2 (ATL2) in the replication of DENV and ZIKV [36,37]. ATL2 is a membrane fusion protein which uses GTP binding and hydrolysis to dimerize across opposing ER membranes, leading to membrane fusion and the formation of three-way junctions in the peripheral ER that help to maintain ER branching and homeostasis [38,39]. Using liposomes in vitro, ATL2 dimers have also been observed to tether juxtaposed membranes without fusing them, though the physiological role of tethering remains under investigation [40–43].

In this study, we define a conserved role for ATL2 membrane remodeling during flavivirus infection. Our results indicate that ATL2 is critical for vRO distribution and organization during replication. We found that ATL2 accumulates at sites of vRO biogenesis, and that its membrane tethering activity was required to facilitate proper viral replication. We further linked the disruption of proper vRO formation in ATL2-depleted cells to increases in immune activation. Finally, we leveraged insights into ATL2 tethering function to design ATL2-acting peptides that limit virus infection. Our results establish ER-to-ER membrane tethering as a critical host process for the spatial organization of flavivirus vROs and demonstrate that this organization is important for viral evasion of cellular immunity. Additionally, our work provides a proof-of-concept for using mechanistic interrogations of host–virus interactions to identify potential therapeutic intervention targets.

## Methods

### Ethics statement

Human PBMCs were obtained from healthy donors in accordance with the Emory University Institutional Review Board (IRB) protocol IRB00045821. All study subjects enrolled in this research provided written informed consent as per protocols approved by the Emory University IRB.

### Viruses and cell lines

Vero E6, HEK-293T, C6/36, and A549 cells were obtained from ATCC. Huh7 cells were obtained from the research group of Dr. Arash Grakoui (Emory University). Huh7-Lunet-T7 cells were obtained from the research group of Dr. Ralf Bartenschlager (University of Heidelberg). All cell lines were regularly tested for mycoplasma contamination; cell lines were authenticated by visual observations of cell morphology. All cells were cultured in Dulbecco’s modification of Eagle’s medium (DMEM, Corning) supplemented with 10% fetal bovine serum, 100 U/mL penicillin, 100 µg/mL streptomycin, and 1% non-essential amino acids (complete media). Stable A549 cell lines were cultured in complete media containing either 5 μg/mL blasticidin or 1 μg/mL puromycin. Huh7-Lunet-T7 cells were cultured in complete media containing 2 μg/mL zeocin.

Molecular clones of DENV genotype 2 (strain 16681), ZIKV (strain H/PF/2013), and the reporter *Renilla* luciferase (RLuc) DENV (strain 16681; DV-R2A) have been previously described and were a gracious gift of Ralf Bartenschlager [44–48]. Isolates of WHO reference strains for DENV serotypes DV1-WP75, DV3-H87, and DV4-H241, as well as CHIKV 181/25 and ONNV UgMP30, were generously provided by Dr. Matthew Collins (Emory University). For molecular clones, virus stocks were prepared as previously described by electroporation of Vero E6 cells with in vitro transcribed viral RNA; harvesting occurred 4–8 days after electroporation [46]. For amplification of isolates or stocks from molecular clones, Vero E6 or C6/36 cells were infected at an MOI of 0.01 and supernatants were harvested 2–8 days after infection, beginning when cells showed cytopathic effect.

Extracellular virus titers were determined by plaque-forming unit (PFU) assay in Vero E6 cells using a minimum essential medium (MEM, Gibco) overlay containing 1.0% carboxymethylcellulose (CMC). For DENV serotypes 1, 3, and 4, titers were determined using a focus-forming unit (FFU) assay; infected cells in MEM-1.0% CMC were fixed and stained for the viral NS3 protein and infection foci were counted using fluorescence microscopy [49]. Titers of the DV-R2A virus, which does not produce plaques on Vero E6 cells, were also calculated using a fluorescence-based approach. Briefly, cells were infected at 10-fold decreasing dilutions by DV-R2A or by a wild-type DENV with a known titer. The percentage of infected cells was determined by immunofluorescence staining for the viral NS3 protein (see below). A sigmoidal regression curve, using the percentage of infected cells caused by the wildtype virus as a standard, enabled quantitation of the R2A virus titer.

### ATL2 expression and depletion constructs

A list of all primers used for ATL2 cloning is provided in S1 Table. For all ATL2 expression constructs, the NEBuilder HiFi cloning system was used as per the manufacturer’s protocol (New England Biolabs). ATL2 mutants were amplified from full-length ATL2-containing plasmids in the pWPI backbone (a gift from Didier Trono, Addgene plasmid #12254); we previously introduced synonymous mutations in the ATL2 gene sequence to remove cryptic bacterial promoters and facilitate plasmid amplification in bacterial strains [36]. Sequences were transferred to the pWPI backbone using the NEBuilder HiFi DNA Assembly Master Mix (New England Biolabs) and subsequently transformed into competent DH5α *Escherichia coli* (Invitrogen). Successful transformants were confirmed using Sanger sequencing (Azenta Life Sciences). Constructs for shRNA-mediated ATL2 depletion, as well as nontarget (NT) shRNA control constructs, have been described previously [36].

### Lentivirus production and transduction of cells

Delivery of expression constructs for depletion and overexpression experiments was accomplished through transduction with lentiviruses. For production of lentiviral stocks, sub-confluent 293T cells were transfected with packaging plasmids pCMV-Gag-Pol (Addgene plasmid #22036) and pMD2-VSV-G (Addgene plasmid #12259), kind gifts from Didier Trono, as well as pWPI insert plasmids. Two days post-transfection, lentivirus-containing media was collected and filtered through 0.45 µm PVDF syringe filters (MilliporeSigma). Lentiviruses were titrated by SYBR Green I-based real-time PCR-enhanced reverse transcriptase (SG-PERT) assay, using the iTaq Universal SYBR Green Supermix (Bio-Rad Laboratories) [50,51]. Lentivirus stocks were compared to a standard curve derived from a sample with a known RNA concentration in order to determine the titer for each stock.

Transductions were performed using an MOI of 5 in the presence of 4 µg/mL polybrene, with the following exceptions. All experiments utilizing glass coverslips for immunofluorescence (see below) did not contain polybrene due to an unknown, previously unobserved, and inexplicable adverse reaction between polybrene-containing media and glass coverslips, which resulted in cell death. Transductions of Huh7 or Huh7-Lunet cells were performed at an MOI of 10 owing to reduced transduction efficiencies in these cells. Stable cell lines were produced by transduction of target cells with lentivirus at an MOI of 1, followed by selection with media containing either 5 μg/mL blasticidin or 1 μg/mL puromycin, depending upon the resistance marker of the lentiviral vector. Viability of transduced cells was evaluated using the CellTiter-Blue Cell Viability Assay (Promega) by measuring fluorescence (excitation 560 nm, emission 590 nm) with a BioTek Synergy H1 multimode microplate reader (Agilent Technologies) according to the manufacturer’s instructions.

### Reverse transcription-quantitative PCR (RT-qPCR)

Cells were washed once with PBS, followed by lysis with RNA Lysis Buffer (Zymo). Total cellular RNA was isolated using a Quick-RNA Miniprep RNA extraction kit (Zymo) according to the manufacturer’s protocol. Synthesis of cDNA was accomplished using a high-capacity cDNA reverse transcription kit (Applied Biosystems) according to the manufacturer’s specifications. After being diluted 1:10, cDNA was used for qPCR analysis, employing specific primers and the iTaq Universal SYBR Green Supermix (Bio-Rad). Primers for qPCR were designed using Primer3 software; primer sequences are supplied in S2 Table. To obtain the relative abundance of specific RNAs from each sample, cycle threshold (Ct) values for cDNAs of interest were normalized to those of hypoxanthine phosphoribosyltransferase 1 (HPRT). All qPCR runs were performed on a C1000 Dx Thermal Cycler with a CFX96 Optical Reaction Module (Bio-Rad). Melting curves were evaluated for each primer set, and samples in which more than one peak was observed were discarded.

### Western blotting

Cells were lysed using 2× Laemmli buffer, followed by sonication and denaturation at 95 °C for 5 min. ~10 µg of total protein from each sample was resolved with a 10% SDS-PAGE gel and transferred to a nitrocellulose membrane. Membranes were blocked with PBS-T (PBS [pH 7.4] with 0.1% Tween-20) containing 5% skim milk for 1 h at room temperature. Primary antibodies were incubated in PBS-T containing 2% skim milk overnight at 4°C. Membranes were washed with PBS-T and incubated for 1 h with the appropriate HRP-conjugated secondary antibodies. For a list of primary and secondary antibodies used, see S3 Table. Following secondary incubation, membranes were again washed with PBS-T and Clarity Western ECL Substrate (Bio-Rad) or Amersham ECL Select Western Blotting Detection Reagent (Cytiva) was applied. Membranes were imaged using the ChemiDoc MP Imaging System (Bio-Rad). Images were cropped and analyzed using Fiji software [52].

### Immunofluorescence (IF)

A549 cells grown on 12 mm glass coverslips (Azer Scientific) or in µCLEAR black-walled 96-well plates (Greiner BioOne) were washed 1× with PBS, fixed with 4% paraformaldehyde at room temperature for 20 min, washed 3× with PBS, and permeabilized in 0.2% Triton X-100 in PBS for 2 min at room temperature. For digitonin permeabilization, instead of Triton X-100, cells were treated with 0.01% digitonin for two minutes at room temperature. Following a wash step (3× with PBS) to remove the permeabilization solution, samples were blocked in 2.5% bovine serum albumin (BSA) or 2.5% skim milk in PBS-0.01% Tween-20 for 1 h at room temperature, then incubated with the indicated primary antibodies (diluted in blocking buffer) at 4 °C overnight (for a list of primary and secondary antibodies used, see S3 Table). Samples were then washed 6 times with PBS-0.01% Tween-20 (3 × 10 s, 3 × 10 min) and incubated with secondary antibodies in PBST for 45 min at room temperature. Following another 6 washes (3 × 10 s, 3 × 10 min), coverslips were mounted onto microscope slides using DAPI Fluoromount-G mounting media (SouthernBiotech). For 96-well plates, DAPI was added at 2.86 µM for 20 min prior to secondary washes.

Epifluorescence images were obtained using an Axio Observer Z1 microscope with an Axiocam 506 monochromatic camera (Zeiss) or a BioTek Lionheart FX automated widefield microscope (Agilent). Confocal images were obtained with a Leica Stellaris 8 Inverted Confocal Microscope (Leica Microsystems) with a white light laser and both HyD-S and Power HyD-X detectors. Image analysis to determine percent infection, fluorescent signal intensity, and fluorescent signal distribution was performed using CellProfiler [53]. Pearson’s correlation coefficients were calculated using the Coloc2 plugin for Fiji. Adobe Photoshop and Illustrator software packages were used to assemble images into figures.

To perform manual quantification of ER reticulation, images from each condition were first blinded by an author uninvolved with image acquisition. Each cell in an image was then classified as either “normally reticulated” or “abnormally reticulated”. Data were then unblinded and results were reported as a percentage of the quantified cells in each condition which exhibited normal reticulation.

### Proximity ligation assays (PLA)

Coverslips were washed twice with PBS, fixed with 4% paraformaldehyde for 20 min at room temperature, and permeabilized with 0.2% Triton X-100 for 20 min. PLA was performed using the Duolink PLA Kit (Millipore-Sigma) according to the manufacturer’s instructions and as previously described [49,54]. Briefly, cells were blocked in the kit buffer for 1 h at 37 °C, then incubated with anti-HA and anti-NS5 primary antibodies for 2 h at room temperature. For a list of antibodies used, see S3 Table. After washing, coverslips were incubated with PLUS and MINUS PLA probes for 1 h at 37 °C, followed by ligation (30 min) and amplification (100 min) at 37 °C. Cells were stained with DAPI (Life Technologies), washed, and mounted using Fluoromount-G (SouthernBiotech). Images were acquired using an LSM780 confocal microscope (Zeiss) at the INRS Confocal Microscopy Core Facility. PLA dot quantification was performed with Fiji software. Signals >0.02 μm^2^ with circularity between 0.02 and 1.00 were counted per cell. Identical acquisition and analysis settings were applied across all experiments.

### Electron microscopy (EM)

Cells grown in 24-well plates were washed with pre-warmed PBS, then fixed for 30 min by incubation with pre-warmed 1.25% glutaraldehyde in 0.2 M HEPES-buffered H_2_O. After 3 washes with 0.2 M HEPES-buffered H_2_O, cells were incubated with 2% osmium tetroxide/0.2 M HEPES-buffered PBS for 40 min on ice. Cells were washed with water 3 times and treated with 0.5% uranyl acetate for 30 min. After a 30-min wash with water, cells were progressively dehydrated with increasing concentrations of ethanol (40%–100%), followed by a wash with propylene oxide and the addition of Epon/araldite resin (Araldite 502/Embed 812 kit; Electron Microscopy Sciences), which was then polymerized at 60 °C for 72 h. Embedded cells were sectioned into 70-nm-thick slices by using an Ultracut UCT microtome (Leica) and a diamond knife (Diatome). After counterstaining with 3% uranyl acetate in 70% methanol for 5 min and 2% lead citrate in water for 2 min, cells were imaged with a JEOL JEM-1400 120kV LaB6 transmission electron microscope (TEM) with a Gatan US1000 CCD camera (Jeol, Tokyo, Japan). Whole-cell overview images at 4,000× or 8,000× magnification were obtained using SerialEM [55].

Quantification of vRO number and location was performed manually in Fiji. Distribution of vROs was quantified using a custom statistical pipeline developed using R and ChatGPT (OpenAI, 2025) [56–60]. All calculated distances were converted from pixels to nm following pipeline completion, using the scale factor 1 pixel = 2.579 nm. For tomography images, 200-nm-thick sections were prepared, and 10 nm-diameter protein A-gold was added to both sides of the grid. Grids were placed in a high-tilt holder, and digital images were recorded as single-axis tilt series over a −60° to +60° tilt range (increment 1°). Tomograms were reconstructed using the IMOD software package (https://bio3d.colorado.edu/imod) and segmented manually using Dragonfly 3D World software (Comet Technologies Canada) [61].

### Correlative light and electron microscopy with immunofluorescence (immuno-CLEM)

Immuno-CLEM was performed as previously described [62,63]. Briefly, A549 cells expressing HA-tagged ATL2 were seeded into glass-bottom cell culture dishes containing etched gridded coverslips (MatTek Life Sciences) and infected with DENV or ZIKV (MOI = 5). After 48 h, cells were fixed in 4% PFA, 0.05% glutaraldehyde, and 0.2 M HEPES in H_2_O for 10 min at room temperature. Cells were washed 2 times with 4% PFA and 0.2 M HEPES in H_2_O, then fixed in this solution for an additional 30 min. After 3 five-minute washes with PBS, cells were incubated with blocking solution (50 mM NH_4_Cl, 0.1% saponin, 1% BSA in PBS) for 30 min. Cells were then incubated with primary antibodies diluted in blocking solution overnight at 4°C (for list of antibodies, see S3 Table). The next day, following 6 two-minute washes with PBS, secondary antibody diluted in blocking solution was applied for 1 h at room temperature. DAPI was then added at 2.86 µM for 15 min. Following 6 more two-minute washes with PBS, cells were imaged using a Leica Stellaris 8 Inverted Confocal Microscope (Leica Microsystems). Z-stacks were obtained and the position of cells of interest on the etched grids was recorded using transmitted light (brightfield). Cells were then fixed and processed for EM as described above. Areas of interest for EM imaging were identified using the MatTek etched grid, visible in the Epon. EM samples were visualized with a JEOL JEM-1400 TEM. The shape of each cell’s nucleus (as indicated by DAPI signal area) was used to correlate and overlay EM images with the appropriate IF Z-slice; alignment was performed using ImageJ and Adobe Photoshop software packages.

### Plasmid-induced replication organelle (pIRO) polyprotein expression system

Huh7-Lunet-T7 cells, which stably express a cytosolic T7 RNA polymerase, were transduced with lentiviruses (MOI 5) containing either NT shRNA or ATL2 shRNA sequences and seeded into 6-well plates. After 2 days, transduced cells were seeded into 24-well plates at 30,000 cells/well. Twenty-four hours later, cells were transfected with either the DENV or ZIKV pIRO construct described previously, using TransIT transfection reagent (Mirus Bio) [64,65]. Eighteen hours after transfection for DENV pIRO, or 16 h after transfection for ZIKV pIRO, cells were processed for EM, IF or western blot as described above. IF was used to evaluate transfection efficiency, as determined by the percentage of cells staining positive for the viral NS3 protein; Western blots were used to verify protein expression level. EM quantification of vROs was performed by systematically surveying cells and evaluating for the presence of vROs, followed by imaging at 8,000× magnification. Only cells with >4 vROs were considered positive. For each condition, >50 cells were surveyed over 3 biological replicates. All observed vROs were imaged, and vRO diameters were determined using Fiji by measuring the distance across two axes and averaging.

### Bulk RNA sequencing (RNA-seq) analysis

A549 cells were transduced with constructs expressing ATL2 shRNA or NT shRNA for 4 days. Four biological replicates were used for each sample. Cells were lysed with RLT buffer (Qiagen) and RNA-seq libraries were prepared at the DLS HudsonAlpha using the NEB Ultra II RNA Library Preparation kit. Libraries were sequenced on a NovaSeq X plus PE100 run. Raw FASTQ reads were mapped to the hg38 human genome using STAR (v2.7.6a) with the GENCODE V27 reference transcriptome [66,67]. Duplicate reads were removed from downstream analysis using the Picard MarkDuplicates (v2.23.8) function (http://broadinstitute.github.io/picard/). Reads mapping to exons for all unique ENTREZ genes were compiled and normalized using GenomicRanges (v1.38.0) and all genes expressed at 3 or more reads per million in all samples of any one biological group were considered expressed [68]. Differential expression analysis was performed with DESeq2 (v1.42.1) and genes with an FDR < 0.05 and absolute log2 fold-change ≥1 were considered significant [69].

### Proteomic analysis

A549 cells expressing lentivirus-delivered ATL2-targeted or NT shRNA for 4 days were scraped and pelleted at 100*g* for 5 min, followed by freezing at −80 °C. Thawed cell pellets were lysed in 250 µL of lysis buffer (10 mM Tris pH 7.0, 150 mM NaCl, 4% sodium dodecyl sulphate [SDS]) containing protease and phosphatase inhibitors (cOmplete and PhosStop, Roche), then sonicated. Clarified protein lysates were precipitated with acetone twice, and 50 µg of normalized protein mixtures was resuspended and denatured in 40 µL U/T buffer (6 M urea/2 M thiourea in 10 mM HEPES, pH 8.0). Proteins were reduced and alkylated in 10 mM DTT and 55 mM iodoacetamide, followed by digestion using 1 µg of LysC (FUJIFILM Wako Chemicals) and trypsin (Promega) in ABC buffer (50 mM NH_4_HCO_3_ in water, pH 8.0) overnight at 25 °C, 800 rpm. After digestion, peptides were purified on stage tips with 3 layers of C18 Empore filter discs (3M) as previously described [70].

Samples were analyzed on a Vanquish Neo LC system (Thermo Scientific) coupled to an Orbitrap Exploris 480 mass spectrometer (Thermo Scientific) equipped with a Nanospray Flex source (Thermo Scientific). Peptides were injected into an Acclaim PepMap 100 trap column (2 cm × 75 μm, 3 μm C18; Thermo Fisher Scientific) and next separated on a 60 cm × 75 μm column (1.7 µm C18 beads UHPLC column; Aurora Ultimate) with a packed emitter tip (Ion Opticks), at a constant flow rate of 300 nL min^−1^ over 120-min linear gradient. The column temperature was maintained at 45 °C using an integrated column oven (Sonation GmbH). The column was equilibrated using 3 column volumes before loading samples in 96% buffer A (99.9% Milli-Q water, 0.1% formic acid [FA]/4% buffer B [99.9% ACN, 0.1% FA]). Samples were separated using a linear gradient from 10% to 31% buffer B over 62 min before ramping up to 43% (33 min), 100% (1 min) and sustained for 10 min, followed by reduction to 4% buffer B (8 min). The Orbitrap Exploris 480 was operated in positive ion mode, with a positive ion voltage of 2,000 V in data-dependent acquisition mode (DDA) using the Thermo Xcalibur software (v. 4.5.474.0). DDA analysis was performed with a cycle time of 1.5 s. Survey scans were acquired at 120,000 resolution, with a full scan range of 350–1,400 *m*/*z*, an automatic gain control (AGC) target of 300% and a maximum ion injection time of 25 ms, intensity threshold of 5 × 10^3^, 2–6 charge state, dynamic exclusion of 90 s, mass tolerance of 10 ppm. The selected precursor ions were isolated in a window of 1.6 *m*/*z*, fragmented by a higher-energy collisional dissociation (HCD) of 30. Fragment scans were performed at 15,000× resolution with an Xcalibur-automated maximum injection time and standard AGC target.

### Raw mass spectrometry data processing and analysis

Raw MS data were processed with the MaxQuant software (v. 2.2.0.0) using the built-in label-free quantitation algorithm and Andromeda search engine [71]. The search was performed against the *Homo sapiens* proteome (UniProtKB release UP000005640; Taxon ID 9606) containing forward and reverse sequences. Additionally, the intensity-based absolute quantification (iBAQ) algorithm and “match between runs” option were used. In MaxQuant, carbamidomethylation was set as fixed and methionine oxidation and N-acetylation as variable modifications. Initial search peptide tolerance was set at 20 p.p.m. and the main search was set at 4.5 p.p.m. Experiment type was set as data-dependent acquisition with no modification to the default settings. Search results were filtered with a false discovery rate of 0.01 for peptide and protein identification. The Perseus software (v. 1.6.15.0) was used to further process the proteomics data. Protein tables were filtered to eliminate identifications from the reverse database and common contaminants. In the subsequent MS data analysis, only proteins identified on the basis of at least one peptide and a minimum of three quantitation events in at least one experimental group were considered. The MaxLFQ protein intensity values were median-normalized and log2-transformed, and missing values were filled by imputation with random numbers drawn from a normal distribution calculated for each sample [72]. Knockdown-specific proteome changes were determined by two-sided Student *t* test (S0 = 1) with permutation-based false discovery rate statistics (250 permutations, FDR threshold 0.05) or using a log2 (fold change) ≥ 2 cutoff. Results were plotted as scatter plots using Perseus [72].

### *Renilla* luciferase assay

The expression of *Renilla* luciferase (RLuc) in cells infected with the DV-R2A reporter virus was used as a surrogate measurement of intracellular viral replication, as described previously [46,73]. Briefly, cells plated in white-walled µCLEAR 96-well plates (Greiner BioOne) and infected with DV-R2A reporter virus for 48 h were lysed in 35 µL of RLuc lysis buffer (0.1% Triton X-100, 25 mM glycine-glycine [pH 7.8], 15 mM MgSO_4_, 4 mM EGTA, 10% glycerol, 1 mM DTT) and freeze-thawed at −80 °C. RLuc activity was measured by adding 100 μl of Rluc assay buffer (15 mM potassium phosphate [pH 7.8], 25 mM glycine-glycine [pH 7.8], 15 mM MgSO_4_, 4 mM EGTA, 1.43 μM coelenterazine) to 15 μl of cell lysate and measuring luminescence using a BioTek Synergy H1 multimode microplate reader.

### Generation of ATL2-KO cells and ATL2-KO rescue experiments

The A549 ATL2 knockout (ATL2-KO) cells used in this work were briefly described previously and were created in the lab of Ralf Bartenschlager [36]. To ensure complete knockout of ATL2 while avoiding clonal effects, we created single-cell clones by plating ATL2-KO cells at a limiting dilution to allow for the growth of single-cell colonies. Individual colonies were tested for ATL2 KO by western blot (S5A Fig), and 5 colonies which displayed complete ATL2 KO across 13 passages were pooled to create a new population of ATL2-KO pooled cells. In parallel, control cells with a nontarget guide sequence were produced using the same method; unaffected expression of ATL2 in control single-cell colonies was also confirmed by western blot (S5A Fig) prior to pooling 5 colonies, creating control pooled cells.

For ATL2-KO rescue experiments with DV-R2A, ATL2-KO pooled cells and control pooled cells were transduced with the specified lentiviruses at an MOI of 5 and plated at 8,500 cells per well into 96-well plates. Twenty-four h after transduction, cells were infected with DV-R2A at an MOI of 1 for 48 h, with media replacement occurring at 24 h post-infection. Viral replication was determined by RLuc assay (see above). Individual experiments contained quadruplicate replicates, and results report all replicates from two independent experiments. For rescue experiments with WT ZIKV, cells were seeded at 40,000 cells per well into 24-well plates and transduced with specified lentiviruses at an MOI of 5. Twenty-four h after transduction, cells were infected with ZIKV at an MOI of 1 for 48 h, with media replacement occurring at 24 h post-infection. Supernatants were collected, then cells were washed once with PBS and lysed with RNA lysis buffer. Viral replication was determined by qPCR and plaque assay (see above). Individual experiments contained two replicates, and results report all replicates from two independent experiments.

### Peptide treatment of infection

A549 cells were seeded at 7,000 cells per well into 96-well plates. The next day, ATL2 and control peptides (Peptide 2.0) and DENV (MOI 1) were added to cells simultaneously. For a list of peptide sequences, see S4 Table. Peptides were applied at 200, 100, 50, or 25 µM concentrations. Media was changed after 1 h, with fresh peptide added (at the same concentrations). Cells were incubated for 48 h, at which point supernatants were harvested for plaque assay and cells were fixed for an IF-based assay to quantify infection levels (as described above). Results in all cases represent a minimum of two replicates from three independent experiments. Viability of peptide-treated cells was evaluated using the CellTiter-Blue Cell Viability Assay (Promega) according to the manufacturer’s instructions.

For peptide experiments in primary human monocyte-derived dendritic cells, moDCs were isolated as previously described [74–76]. Briefly, whole blood was drawn from three anonymized donors and deposited into Vacutainer CPT tubes (BD Biosciences). Monocyte separation proceeded according to the manufacturer’s instructions. CD14 cells were isolated using the MojoSort Human CD14 Selection Kit (BioLegend) as per the manufacturer’s instructions. These cells were resuspended in RPMI 1640 (Corning) supplemented with 10% FBS, 2 mM L-glutamine, 1 mM HEPES, 1 mM sodium pyruvate, 1% non-essential amino acids, 100 U/mL penicillin, and 100 µg/mL streptomycin (complete RPMI). Granulocyte-macrophage colony-stimulating factor (GM-CSF) and interleukin-4 (IL-4) were also added to the media at 100 ng/mL each, and cells were plated into 6-well plates. One day after plating, cells were fed with fresh complete RPMI and cytokines. Five days after plating, non-adherent cells (differentiated moDCs) were collected and resuspended in complete RPMI, then plated at 100,000 cells/well into V-bottom 96-well plates. Cells were treated with the indicated peptide and infected with DENV or ZIKV (MOI 2), as above. Supernatants were harvested from each well at 24 and 48 h post-infection. At 48 h post-infection, cells were lysed for total RNA isolation. Viral titers were determined by plaque assay and viral RNA levels by RT-qPCR using the methods described above. Results represent duplicate samples from three different donors. Viability of treated cells was evaluated using the CellTiter-Blue Cell Viability Assay.

### Statistical analyses

Except for the mass spectrometry and RNA-seq data described above, all statistical analyses were performed with GraphPad Prism software (v11.0.2). For experiments in which comparisons were made between only two conditions, unpaired two-tailed Welch’s *t*-tests were used to compare means when sample data were normally distributed, while Mann–Whitney tests were used to compare ranks for experiments in which sample data were not normally distributed. For experiments in which comparisons were made between three or more independent conditions, Welch’s one-way ANOVA with Dunnett’s T3 test was employed to compare means of normally distributed sample data, while a Kruskal–Wallis test was used to compare ranks for sample data which were not normally distributed. For experiments investigating the effects of two different independent variables (and resulting comparisons between conditions), a two-way ANOVA with Tukey’s Honestly Significant Difference (HSD) was used to compare means. In all cases, the threshold for significance (alpha) was set to 0.05. Unless otherwise stated, error bars show standard error of the mean (SEM).

## Results

### ATL2 depletion alters the intracellular distribution of viral replication sites for diverse flaviviruses

**Fig 1:**
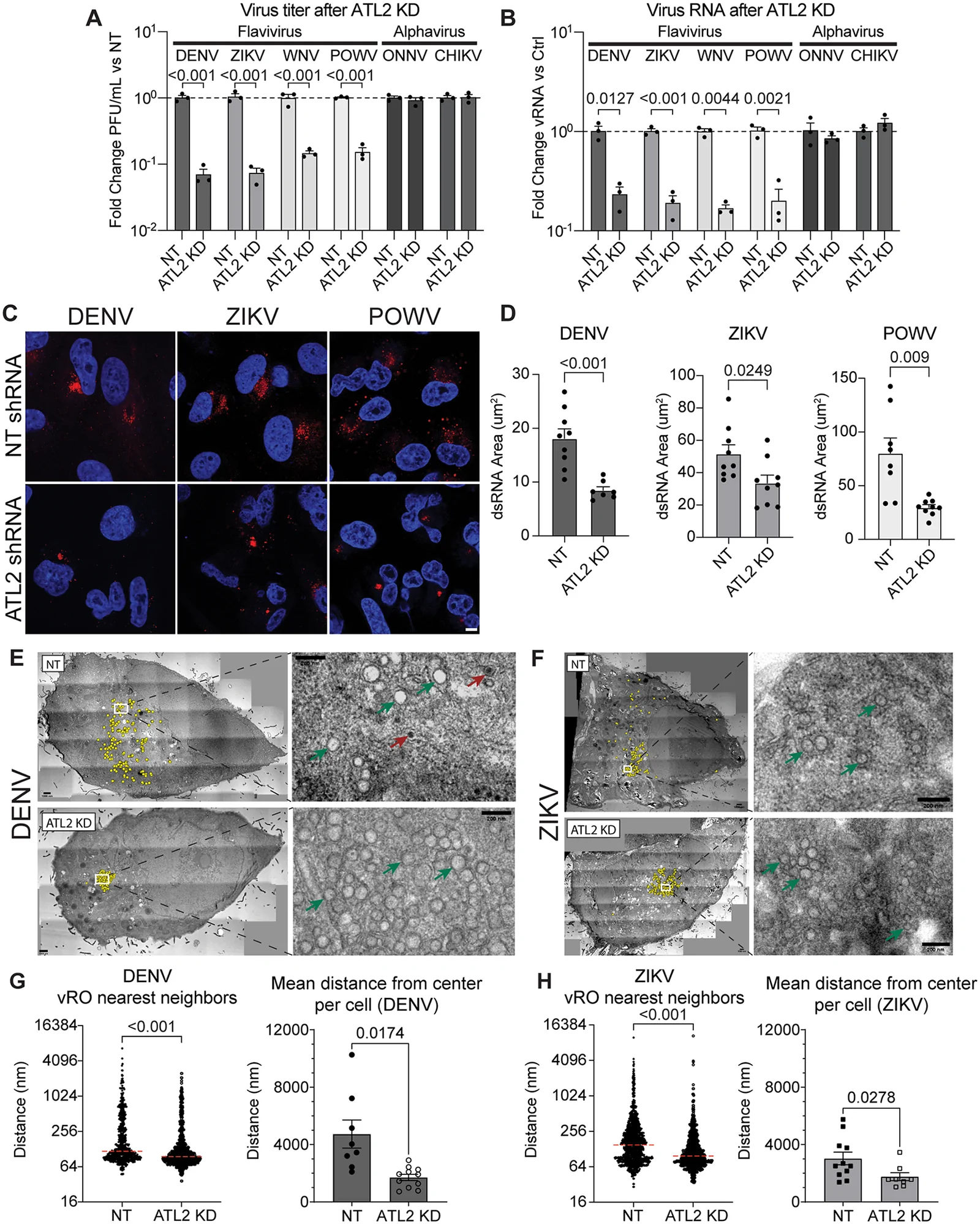
Impact of ATL2 depletion on flavivirus replication. A549 cells were transduced with lentiviruses encoding either nontargeting shRNA (NT) or shRNA specific for ATL2 (ATL2 KD). Forty-eight h after transduction, cells were infected with the indicated virus. **(A and B)** Transduced cells were infected with DENV2 (strain 16681), ZIKV (strain H/PF/2013), POWV (strain Spooner), ONNV (strain UgMP30) or CHIKV (strain 181/25) for 48 h, followed by evaluating virus titer (**A**) and intracellular viral RNA **(B)**. **(A)** Graphs showing the mean fold change in PFU/mL relative to the NT condition; *n* = 3 biological replicates. **(B)** Graphs showing the mean fold change in viral RNA levels relative to the NT condition; *n* = 3 biological replicates. For **(A and B)**, statistical significance was determined using Welch’s *t* test comparing NT and ATL2 KD conditions for each virus; results of each *t* test are displayed simultaneously. **(C and D)** Cells were fixed with PFA and viewed by immunofluorescence microscopy using antibodies of given specificities. **(C)** Images are representative of 3 independent experiments. Scale bar = 5 μm. **(D)** Graphs show the average area occupied by dsRNA signal in each cell, per image. **(E and F)** NT and ATL2 KD cells were infected with DENV (panels **E, G**) or ZIKV for 24 h (panels **F, H**) and viewed by TEM. **(E and F)** Representative TEM images of infected cells. Viral replication organelles (vROs) were identified manually, with vRO locations labeled with yellow dots. Insets display magnified views of the boxed area; vROs are labeled with green arrows and virions are indicated by red arrows. **(G and H)** Centrographic statistics were used to quantify vRO distributions in NT or ATL2 KD cells infected with either DENV or ZIKV. Left graph in each panel shows the distance between all vROs and their nearest neighbor (red line indicates median value). Right graph in each panel shows the mean distance from center of the vRO distribution for each cell. Statistical significance was determined using a Mann-Whitney test for nearest neighbor data and Welch’s *t* test for mean distance from center data; *n* > 650 vROs per condition. For all graphs, error bars show standard error of the mean (SEM). The data underlying this Figure can be found in S1 Data.

We first aimed to determine if the organization of membrane replication sites by the host GTPase ATL2 was conserved among flaviviruses and for other positive-strand RNA viruses [36]. The reported cellular function of ATL2 as an ER-shaping protein led us to hypothesize that ATL2 depletion would broadly negatively impact flavivirus infections while minimally impacting alphaviruses, which primarily use the plasma membrane or endosomal/lysosomal membranes for replication [77,78]. To test this, ATL2 was depleted in human A549 cells using lentivirus-transduced ATL2-targeted short hairpin RNA (shRNA), and cells were infected with representative flaviviruses or alphaviruses. Cells were monitored for viability, intracellular genome replication, and viral titers. Results from qPCR and plaque assays showed a significant decrease in viral titers and intracellular viral genome levels for all flaviviruses tested – including all four serotypes of DENV as well as ZIKV, WNV, and POWV – in ATL2 knockdown (ATL2 KD) cells, as compared to cells transduced with a nontarget (NT) shRNA construct (Figs 1A, 1B, and S1A–S1C). Conversely, we observed that ATL2 KD did not significantly reduce genome replication in cells infected with the alphaviruses O’nyong’nyong virus (ONNV) and chikungunya virus (CHIKV) (Fig 1A and 1B). Importantly, cell viability analysis showed that depletion of ATL2 did not have a detrimental effect on cell growth that would generally limit virus replication and assembly (S1D Fig). Together, these results indicate that the requirement for ATL2 is conserved for flaviviruses that replicate on ER membranes, and that ATL2 does not have a significant role in alphavirus replication, which occurs primarily at the plasma membrane.

We next evaluated the effect of ATL2 depletion on the localization of flavivirus replication sites, using viral double-stranded RNA (dsRNA, a viral replication intermediate) as a marker for areas of viral genome replication. ATL2-depleted cells infected with either DENV, ZIKV, or POWV were stained for dsRNA and imaged using confocal microscopy. In flavivirus-infected ATL2 KD cells, we observed that dsRNA was primarily localized to a single punctum in the perinuclear region, exhibiting a reduced total area per cell compared to cells expressing nontarget (NT) shRNA (Fig 1C and 1D). These results showed that ATL2 depletion affects the spatial organization of replication sites across three diverse flaviviruses, suggesting that the mechanism of ATL2 function during flavivirus infection is conserved.

Since dsRNA forms as a replication intermediate inside vROs, the change in distribution of dsRNA indicates that vRO organization is likely altered in ATL2-depleted cells [79]. To determine the impact of ATL2 KD on vRO organization, we compared vRO distributions in ATL2 KD and NT cells using transmission electron microscopy (TEM) (Fig 1E–1H). Tiled images of whole cells were used to manually identify vRO locations (Fig 1E and 1F). Centrographic statistics were used to describe vRO distributions in each cell. For both DENV and ZIKV, ATL2 KD significantly decreased both the distance between a given vRO and its nearest neighbor, as well as the mean distance from center for a given cell’s vRO distribution, as compared to NT cells (Fig 1G and 1H). Decreases were also noted in the average nearest neighbor distance per cell (i.e., the average distance between a vRO and its closest neighbor, within each cell) and standard distance per cell (i.e., the dispersion of vROs around the mean center of their distribution, for each cell) (S1E–S1J Fig). Taken together, these results demonstrate that ATL2 depletion alters the spatial organization of vROs for multiple flavivirus infections, causing vROs to form closer together and concentrate around the center of their distribution.

### ATL2 depletion disrupts vRO formation without disrupting protein production

**Fig 2:**
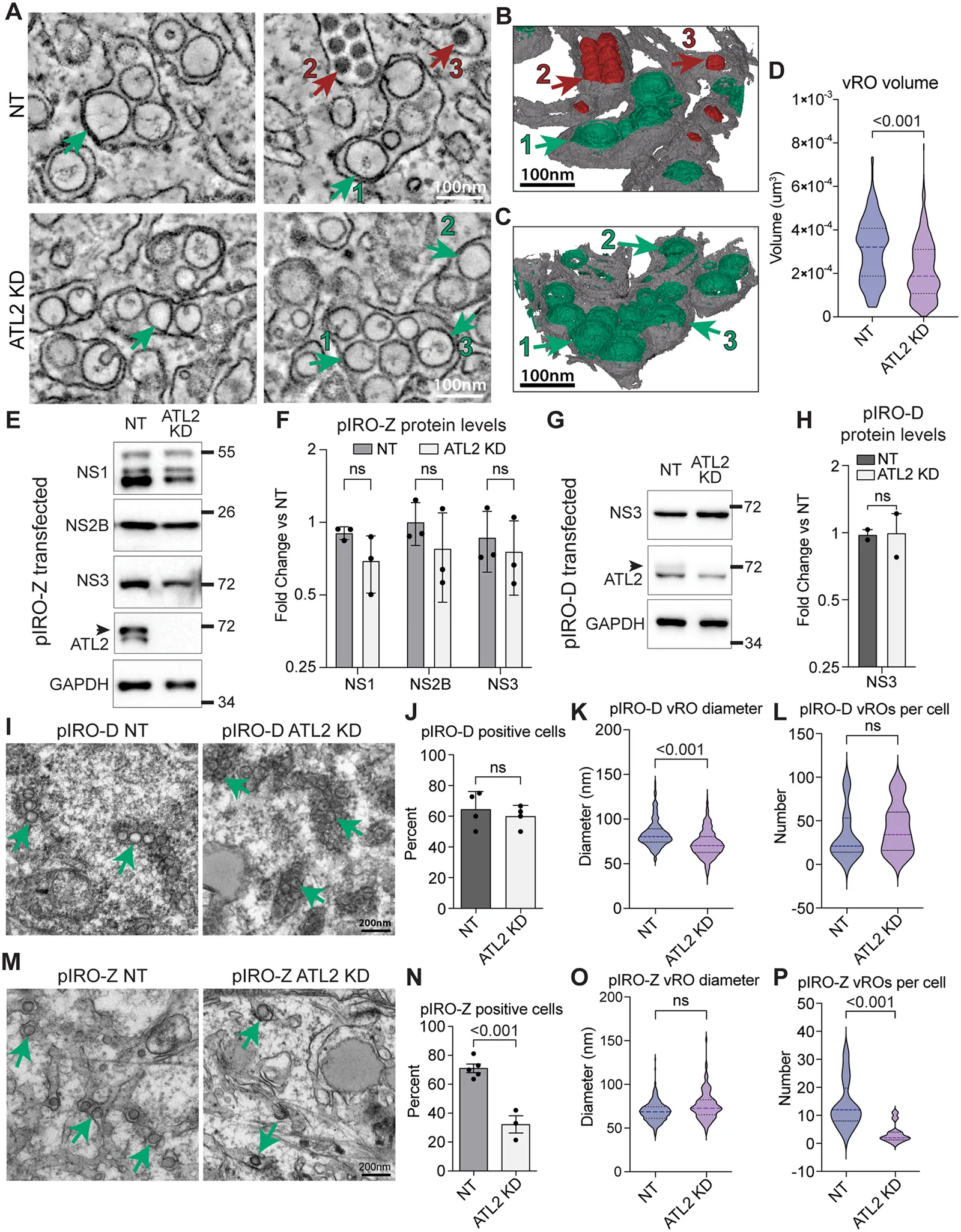
ATL2 depletion does not impact cellular or viral translation. **(A–D)** Cells were transduced with constructs encoding for shRNA directed against ATL2 (“ATL2 KD”), or a nontargeting shRNA (“NT”). Forty-eight h after transduction, cells were infected with DENV for 48 h and analyzed by electron tomography. **(A)** Representative single slices of reconstructed tomograms. Two images are shown for NT shRNA and ATL2 shRNA. Arrows mark vROs (green) or virions (red). In the rightmost image, arrows have been numbered to indicate vROs and virions corresponding to those reconstructed in **B** and **C**. **(B and C)** Reconstruction and segmentation of tomograms for area shown in rightmost images of **A**. **(D)** Graph shows the average total volume of vROs from reconstructed tomograms; *n* ≥ 250 vROs per condition. **(E–P)** Lunet-T7 cells were transduced with ATL2 or NT shRNA for 48 h, followed by transfection with either pIRO-Z (**E, F** and **M–P**) or pIRO-D (**G, H** and **I–L**) constructs. **(E–H**) Cells were lysed and viral protein levels evaluated using Western blotting with the indicated antibodies. **(F, H)** Graphs show the average fold change in protein levels, as compared to NT conditions; *n* = 3 biological replicates for (**F**) and 2 biological replicates for **(H)**. **(I, M)** Representative EM images for pIRO-Z- and pIRO-D-expressing cells, with vROs labeled by green arrows. **J–L** and **M–P**, Graphs show the average percentage of cells that contain vROs **(J, N)**, the average vRO diameter (**K, O**) and the average number of vROs per positive cell **(L, P)**; *n >* 40 cells for >3 biological replicates. Statistical significance was determined by Welch’s *t* tes*t* (**F, H, J, N**) or Mann–Whitney test (**D, K, L, O, P**) comparing ATL2 KD to NT for each sample; ns = not significant. Error bars in all graphs depict SEM. The data underlying this Figure can be found in S1 Data.

To further evaluate the role of ATL2 in vRO biogenesis, we next measured the size of vROs from ATL2-depleted cells. We previously reported that ATL2 depletion altered the diameter of DENV vROs [36]. However, these data were obtained from EM thin sections which cannot accurately depict three-dimensional (3D) morphology. To determine if ATL2 KD resulted in an overall decrease in the 3D volume of vROs, we used electron tomography on DENV-infected NT or ATL2 KD cells (Fig 2A–2C). Evaluation of 3D-reconstructed vROs from NT cells showed an average volume of 3.1 × 10^−4^ μm^3^, consistent with previous reports for DENV infection (Fig 2D) [18]. In ATL2 KD cells, vROs had a significantly lower total volume of 2.16 × 10^−4^ μm^3^, indicating that ATL2 may play a direct role in vRO formation.

The formation of flavivirus vROs requires the accumulation of several viral non-structural proteins as well as specific RNA structures in the viral genome [65]. Therefore, it is possible that the observed changes in vRO organization following ATL2 depletion could result from decreases in viral protein translation or polyprotein processing. To determine if ATL2 depletion leads to global changes in cellular translation, we first compared total mRNA and protein levels between uninfected NT and ATL2 KD cells by integrating bulk RNA-seq and quantitative proteomics (S2A–S2E Fig). Comparisons of differentially regulated transcripts and proteins identified a group of 103 host targets consistently upregulated at both the mRNA transcript and protein level, as well as 115 host targets consistently downregulated at both the transcriptional and translational levels, in ATL2 KD cells (S2A Fig). Among the genes significantly upregulated following ATL2 depletion, we noted a slight functional enrichment for factors involved in inflammatory responses and TNF signaling (S2A and S2D Fig). To specifically investigate possible selective effects of ATL2 on cellular translation, we additionally analyzed host proteins displaying altered abundance in the absence of significant changes at the mRNA level. This approach revealed only a small number of up- or down-regulated proteins in ATL2 KD cells when compared to NT controls, suggesting a lack of translation shut-off in ATL2 KD cells (8 and 18 proteins up- or down-regulated, respectively) (S2B and S2C Fig). Additionally, gene set enrichment analysis of upregulated or downregulated factors using curated “Hallmark” pathways did not show an enrichment for proteins involved in translation in either the transcriptional or proteomic data sets (S2D and S2E Fig) [80]. Taken together, these results indicate that ATL2 depletion dysregulates expression of selected cellular genes at the mRNA and protein levels but is not associated with a general shutoff of host translation.

To determine if ATL2 selectively modulates viral protein synthesis or stability, we employed the plasmid-induced replication organelle (pIRO) system, which expresses flavivirus sub-genomic RNA under the control of an exogenous T7 promoter. Upon transfection of the DENV (pIRO-D) or ZIKV (pIRO-Z) pIRO constructs into Huh7-Lunet cells expressing a cytosolic T7 RNA polymerase (Lunet-T7 cells), viral nonstructural proteins are constitutively expressed, leading to the formation of vROs (S2F Fig) [64,65]. Thus, this system allows for specific evaluation of viral protein production and vRO formation outside the context of virus replication. To determine the impact of ATL2 KD on viral protein production, Lunet-T7 cells were transduced with lentiviruses expressing ATL2 or NT shRNA, followed by transfection with either pIRO-D or pIRO-Z. Immunofluorescence staining for viral NS3 and imaging of transfected cells showed that ATL2 depletion did not alter the transfection efficiency of pIRO-D or pIRO-Z (S2G and S2H Fig). Western blot analysis showed that production of viral non-structural proteins was not significantly different between control and ATL2-depleted cells, indicating that ATL2 depletion does not specifically impair viral polyprotein production (Fig 2E–2H). To determine if ATL2 depletion altered vRO biogenesis in the absence of viral replication, we evaluated pIRO-transfected NT and ATL2 KD cells by TEM (Fig 2I–2P). Paralleling results from infected cells, ATL2 depletion in cells transfected with the dengue-specific pIRO-D caused clustering of vROs and reductions in vRO diameters compared to NT control cells (Fig 2I–2L). Interestingly, in cells transfected with the Zika-specific pIRO-Z, vRO clustering was not observed during ATL2 depletion (Fig 2M–2P); however, we did see a significant reduction in the number of cells containing vROs, indicating a decrease in the efficiency of vRO production in this expression system. Together, these results indicated that ATL2 depletion caused a defect in proper vRO formation even in the absence of viral genome replication, and that this defect is not due to changes in viral protein levels.

### ATL2 depletion increases interferon-stimulated gene activation in flavivirus-infected cells

**Fig 3:**
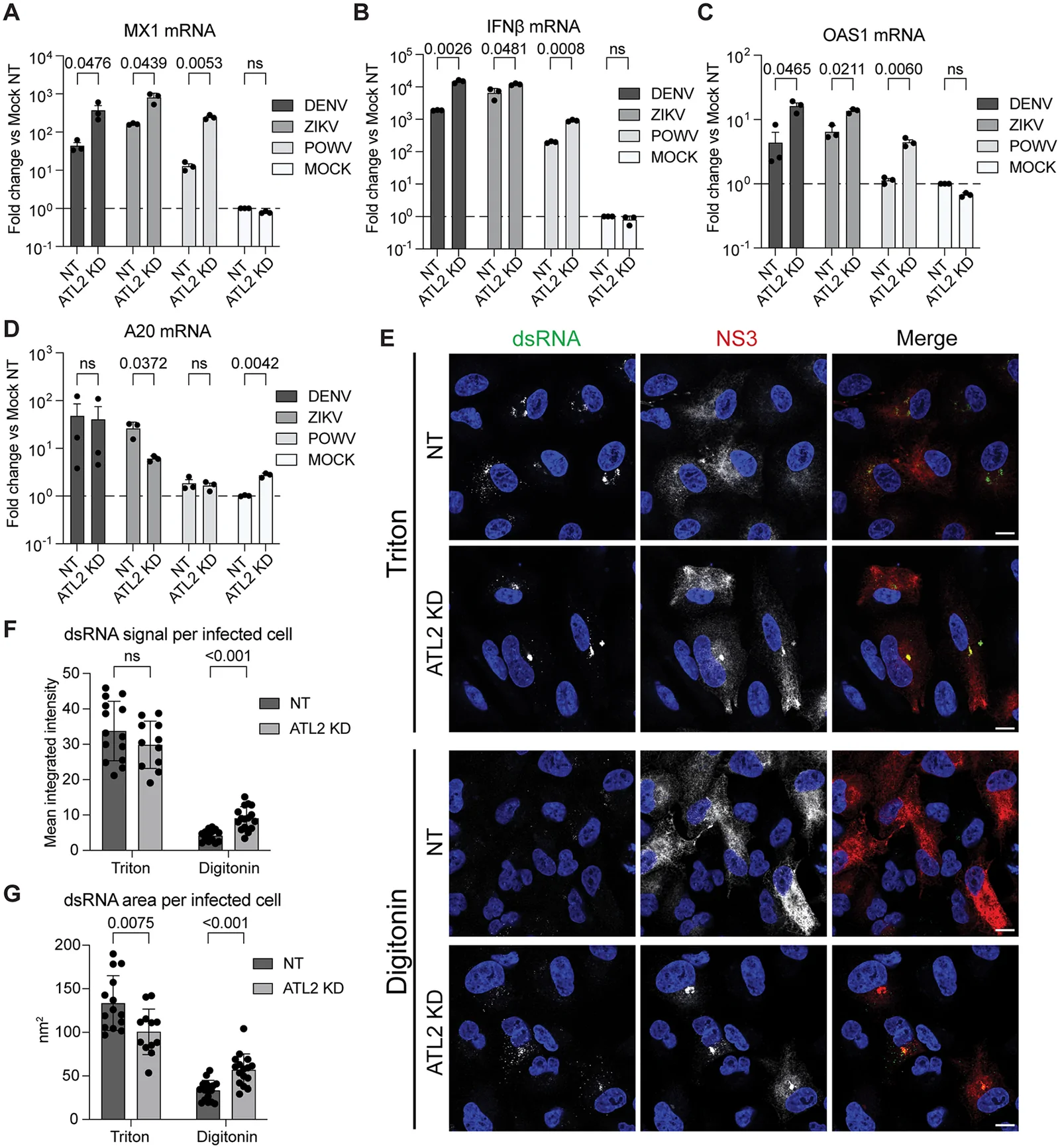
ATL2 KD alters immune gene activation in flavivirus-infected cells. Cells were transduced with lentiviruses encoding for shRNAs directed against ATL2 (“ATL2 KD”) or a NT shRNA (“NT”). **(A–D)** 48 h after transduction, cells were infected with DENV, ZIKV, POWV or mock-infected for 24 h, followed by evaluation of mRNA levels of the indicated genes by RT-qPCR. Graphs show the mean fold change in each transcript level compared to mock-infected NT cells. **(E–G)** Cells transduced with constructs encoding for NT shRNA or ATL2 shRNA were infected with DENV for 24 h, then fixed and permeabilized with either 0.2% Triton X-100 (top) or 0.01% digitonin (bottom). Cells were immunostained for dsRNA and viral NS3, then imaged via confocal microscopy. Representative images are shown in **E**, with quantification in **F** and **G**; graphs show the average integrated intensity of dsRNA signal per infected cell **(F)** or average total dsRNA signal area per infected cell **(G)**. Statistical significance was determined using Welch’s *t* test, comparing NT to ATL2 KD for each virus or condition. Results of each individual test are displayed simultaneously; ns = not significant. Error bars in all graphs depict SEM. The data underlying this Figure can be found in S1 Data.

A primary predicted function of vROs is to conceal viral RNA replication intermediates from sensing by cellular PRRs, including RIG-I and MDA5 [17,21,22]. We therefore reasoned that altered vRO formation and organization in ATL2 KD cells might lead to increased exposure of viral RNA to PRRs. To test this, we evaluated the levels of innate immune genes in ATL2-depleted cells infected with flaviviruses. Interestingly, despite the observed reduction in viral replication, we observed a significant increase in interferon beta (IFN-β) and the interferon-stimulated genes (ISGs) MX1 and OAS1 in ATL2 KD cells (Figs 3A–3C, S3A, and S3B). Similar increases were not observed for the TNF-activated gene A20, although we noted a slight increase in A20 levels in ATL2 KD mock-infected cells, which was consistent with an enrichment for TNF signaling and inflammatory response genes observed by RNA-seq (Figs 3D and S2D). To rule out the possibility that ATL2 depletion generally primes cells for increased interferon activation, we also evaluated immune gene activation in alphavirus-infected cells. During ONNV or CHIKV infection, we observed ISG activation, but there was no significant difference between NT and ATL2 KD cells for the genes we investigated (S3C–S3F Fig), further suggesting a specific mechanism of ATL2 in flavivirus-induced innate immune responses to infection. These data showed that ATL2 depletion leads to increased immune activation specifically in flavivirus-infected cells.

The observed increase in ISG mRNA levels following ATL2 depletion in flavivirus-infected cells could indicate that vRO perturbation caused by ATL2 KD increases access to viral RNA replication intermediates, including dsRNA. To determine if ATL2 depletion leads to increased accumulation of cytosolic dsRNA in flavivirus-infected cells, we evaluated dsRNA fluorescence signal in cells permeabilized with digitonin, which permeabilizes the plasma membrane but not intracellular membranes, limiting antibody access to the interior of vROs [81–83]. As expected, when compared to cells treated with Triton X-100 (which permeabilizes all cellular membranes, including the ER), digitonin permeabilization resulted in significantly decreased dsRNA signal in both NT and ATL2 KD cells, indicating that most viral dsRNA was protected from antibody recognition (Fig 3E). However, in ATL2 KD cells, we observed an increase in the total amount of dsRNA signal, as well as an increase in the total area of that signal, compared to NT cells following digitonin permeabilization (Fig 3E–3G). These results suggest that, upon ATL2 depletion, there is increased dsRNA in the cytosol, consistent with increased recognition by cellular RIG-I-like receptors (RLRs) during infection.

Increased innate immune activation and cytosolic dsRNA levels in flavivirus-infected ATL2-depleted cells suggested that antiviral interferon responses could be the primary driver of decreased viral replication in ATL2 KD cells, rather than a direct impact of vRO perturbation on genome replication. To test this, we performed a knockdown of both ATL2 and the mitochondrial antiviral signaling protein (MAVS), a key signaling protein for both MDA5- and RIG-I-mediated innate immune activation during flavivirus infection [84–88]. If interferon responses cause decreased viral replication during ATL2 depletion, concomitant MAVS depletion should restore viral replication to the level seen in NT cells. As expected, depletion of MAVS increased DENV and ZIKV replication (S3G and S3H Fig) [86,89]. In MAVS-KD/ATL2-KD cells, viral RNA levels remained significantly lower than in MAVS-KD cells; however, the observed differences in viral RNA levels between NT and ATL2-KD conditions were not as pronounced in double KD cells compared to single KD. This difference is consistent with a decrease in the efficiency of ATL2 depletion in cells treated with both MAVS and ATL2 shRNA, but could also indicate that reduced viral replication in ATL2-depleted cells is in part due to increased immune activation (S3I Fig). Importantly, MAVS RNA levels were efficiently reduced in all conditions (S3J Fig) and double-depletion of MAVS and ATL2 did not appreciably reduce cell viability (S3K Fig). Together, these results indicated that even with impaired activation of interferon responses, ATL2 depletion still limited flavivirus genome replication. Thus, while increased immune activation may have some impact on virus growth, our results still support a direct role for ATL2 in facilitating viral genome replication.

### ATL2 is enriched in cellular regions containing vROs

**Fig 4:**
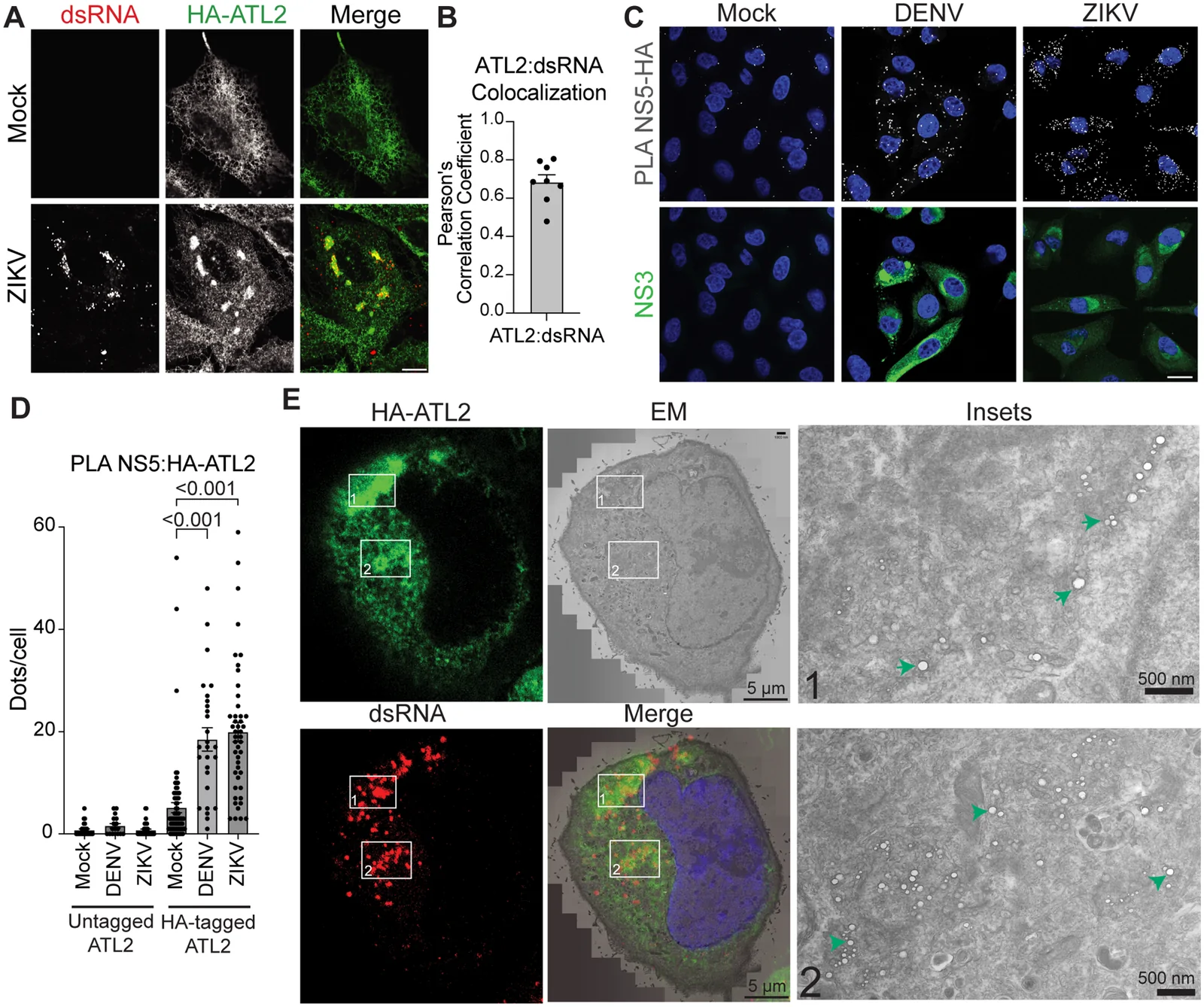
ATL2 colocalizes with sites of vRO formation during flavivirus infection. **(A–D)** A549 cells expressing HA-tagged ATL2 were infected with DENV or ZIKV. **(A)** 24 h after ZIKV infection, cells were fixed and stained for dsRNA and HA using specific antibodies. Panel shows representative images for mock-infected and ZIKV-infected cells. Scale bar = 5 µm. **(B)** Graph shows the average Pearson’s correlation coefficients for the indicated fluorescence signals for 8 images (*n* ≥ 30 cells). **(C, D)** 24 hours after infection with ZIKV, or 48 h after infection with DENV, cells were fixed and subjected to proximity ligation assays (PLA) using anti-HA and anti-NS5 antibodies to detect HA-ATL2:NS5 complexes, and immunostained for the viral NS3 protein to identify infected cells. Cells were imaged using confocal microscopy. Representative images are shown. Scale bar = 20 µm. **(D)** Quantification of PLA dot abundance for HA-ATL2:NS5 interactions in uninfected and infected cells from two independent experiments. Graph depicts the average number of PLA dots per cell. Statistical significance was quantified using a Kruskal–Wallis test. **(E)** A549 cells expressing HA-tagged ATL2 were infected with ZIKV. After 48 h, cells were fixed with paraformaldehyde and glutaraldehyde, followed by staining for dsRNA and HA using specific antibodies and reversible saponin permeabilization. Cells were imaged by confocal microscopy, then immediately embedded and processed for EM imaging. Correlation of fluorescent and EM data was performed with Fiji and Adobe Photoshop software. Numbered insets are a magnification of the corresponding numbered boxed areas. Several individual vROs are marked with green arrows in the insets. For all graphs, error bars depict SEM. The data underlying this Figure can be found in S1 Data.

We next aimed to determine if ATL2 was associated with sites of flavivirus replication. A549 cells expressing HA-tagged ATL2 (HA-ATL2) were infected with DENV or ZIKV, or mock-infected, and the localization of ATL2 and viral proteins/dsRNA was evaluated by immunofluorescence and confocal microscopy. In uninfected cells, ATL2 fluorescence signal was spread in a reticular pattern throughout the cytoplasm, typical of ER localization, as previously reported (Fig 4A) [38,90]. However, in both ZIKV- and DENV-infected cells, ATL2 signal was enriched at sites containing both dsRNA and NS3 signal (Figs 4A, 4B, S4A, and S4B). General ER concentration has been reported at sites of flavivirus replication, but interestingly, co-stains of dsRNA and the ER resident protein reticulon-3 (RTN3) did not show similar co-localization, suggesting that ATL2 or specific ATL2-containing ER microdomains are recruited to sites of flavivirus genome replication (S4C Fig) [25,26].

To further confirm the proximity of ATL2 to sites of virus replication, we used proximity ligation assays (PLA). This technique determines whether two proteins are associated within cells (at a maximum distance of 40 nm) and further permits the localization of intracellular protein complexes by confocal microscopy. PLAs were performed in DENV-, ZIKV- or mock-infected cells expressing HA-ATL2, evaluating the proximity of HA-ATL2 to viral nonstructural protein 5 (NS5, the viral RNA-dependent RNA polymerase). As an additional specificity control, we also evaluated PLA signal in cells expressing untagged ATL2. In HA-ATL2 cells infected with either DENV or ZIKV, we observed a significant increase in PLA signal for HA-ATL2:NS5, compared to mock-infected or untagged ATL2 (Figs 4C, 4D, and S4D). Since cytosolic NS5 is primarily localized to vROs, these results indicate that ATL2 is enriched in cellular regions proximal to vROs [18].

To demonstrate that areas enriched for ATL2 fluorescence signal contain vROs, we employed correlative light and electron microscopy with immunofluorescence (immuno-CLEM), which combines confocal imaging with TEM [62,63,91]. Cells expressing HA-ATL2 were infected with ZIKV for 48 h, fixed, immunostained, and imaged using confocal microscopy, followed by processing for TEM imaging. Overlaying confocal and TEM images, we found that regions enriched for ATL2 and dsRNA signal correlated well with regions containing vROs (Fig 4E). Indeed, vROs were only observed in areas of enriched ATL2 signal. These results confirmed that ATL2 is present at the sites of vRO formation during flavivirus infection, consistent with a direct role in vRO biogenesis.

### ATL2 membrane tethering is critical for flavivirus replication

**Fig 5:**
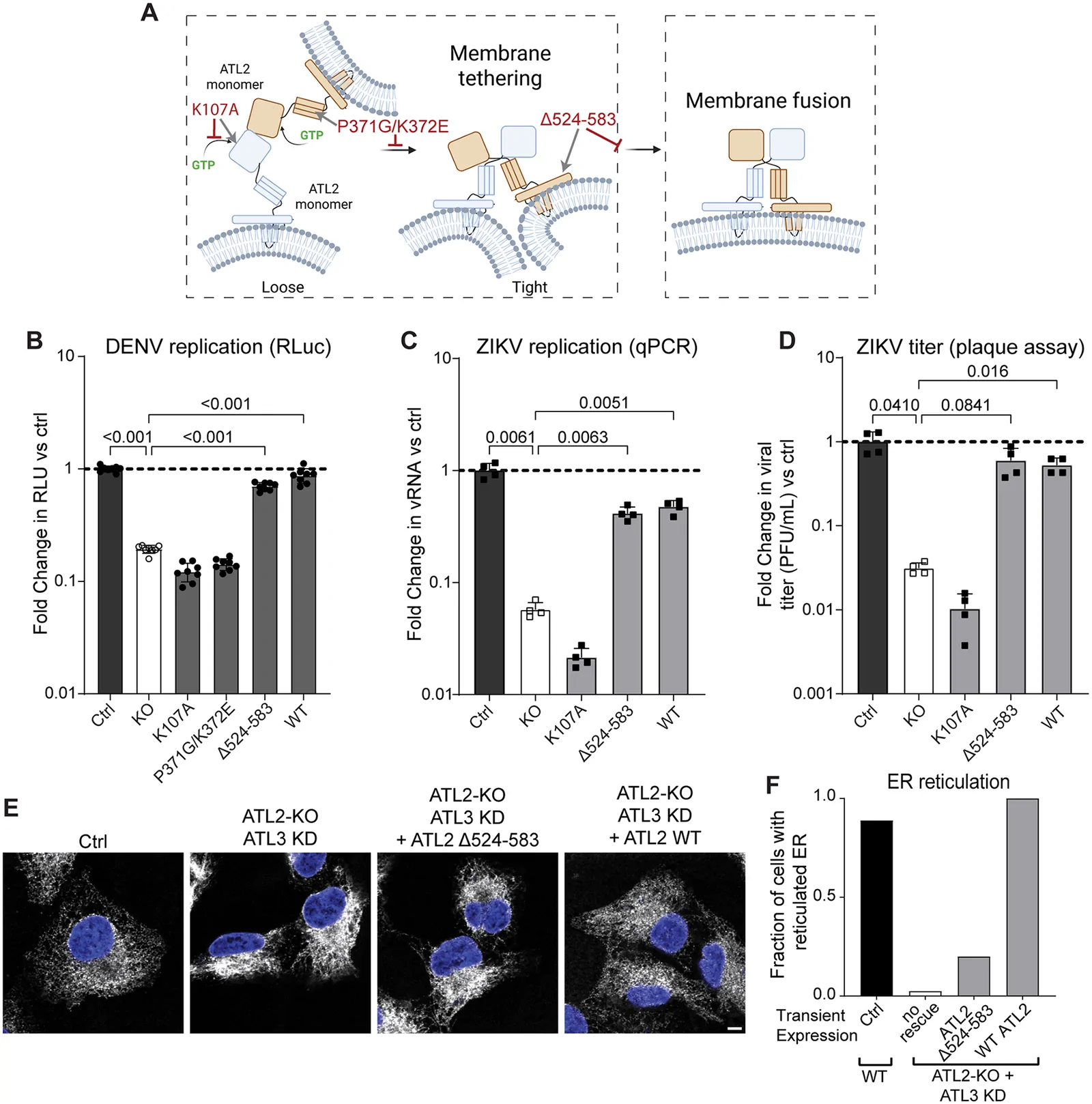
Membrane tethering-competent ATL2 variants rescue flavivirus replication in ATL2-KO cells. **(A)** Schematic of the membrane tethering and fusion mechanism of ATL2, showing ATL2 mutation sites (gray arrows) and effects on tethering and/or fusion (red inhibitors). Citation to Use: Created in BioRender. Neufeldt, C. (2026) https://app.biorender.com/citation/6a03385b42b2c929c3b8d60f. **(B–D)** Flavivirus replication was assessed in control (Ctrl) and ATL2 knockout (KO) cells, as well as in ATL2 KO cells rescued by exogenous transient expression of the designated ATL2 mutants. **(B)** Graph shows the average fold change in *Renilla* luciferase (RLuc) signal from replication of RLuc-containing DENV. **(C)** Graph shows the average fold change in intracellular viral RNA from ZIKV. **(D)** Graph shows the average fold change in ZIKV titers (PFU/mL, measured by plaque assay). For **(B–D)**, results were normalized to viral replication in control cells expressing an empty lentiviral vector (left bar in each graph). Statistical significance was determined by one-way ANOVA with Dunnett’s multiple comparison analysis. **(E, F)**, ATL2 KO cells were transduced with lentivirus constructs expressing ATL3-targeted shRNA for 48 h, then transduced with constructs expressing either ATL2 Δ524–583 or WT ATL2. After another 48 h, ER morphology was assessed by immunostaining for protein disulfide isomerase (PDI). **(E)** Representative images display PDI signal in gray and DAPI in blue. For comparison, control cells and ATL2-KO/ATL3 KD cells transduced with empty vectors are included at left. Scale bar = 5 µm. **(F)** Manual quantification of cells with properly reticulated ER was performed. Graph shows the proportion of cells in each condition which displayed normal ER morphology; *n* ≥ 15 cells and 3 images per condition. For all graphs, error bars show SEM. The data underlying this Figure can be found in S1 Data.

Atlastin-mediated membrane fusion has several well-characterized steps: following GTP binding, ATL2 monomers dimerize across membranes (loose membrane tethering), with a subsequent conformational change bringing membranes into closer association (tight tethering) and leading to eventual membrane fusion. To determine the specific function of ATL2 that facilitates flavivirus infection, we leveraged previously defined ATL2 mutations that block specific steps of this process: K107A (blocks dimerization), P371G/K372E (blocks tight tethering and fusion), and Δ524–583 (blocks fusion) (Fig 5A) [38,41,92–100]. ATL2 mutants were expressed in control or ATL2-knockout (ATL2-KO) cells (see Methods and S5A Fig), followed by flavivirus infection and evaluation of viral replication. Expression of all mutants was verified by western blot and did not impact cell viability (S5B and S5C Fig).

In cells infected with *Renilla* luciferase-expressing DENV (DV2-R2A), expression of full-length wildtype (WT) ATL2 restored DV-R2A replication in ATL2-KO cells to levels comparable to control cells (Fig 5B). Importantly, overexpression of WT ATL2 did not significantly alter DV-R2A replication in control cells (S5D Fig). Expression of the GTP-binding mutant ATL2 K107A, which inhibits ATL2 dimer formation, was unable to restore DV-R2A replication in ATL2-KO cells, indicating that ATL2 GTP nucleotide binding is required for flavivirus replication. Interestingly, expression of this mutant had a slight negative impact on viral replication in control cells, likely through a previously documented dominant-negative effect [38,98]. Similarly, expression of ATL2 P371G/K372E, which is capable only of loose membrane tethering, did not rescue virus replication in ATL2-KO cells and reduced viral replication in control cells [92,98]. However, expression of a mutant which is missing the C-terminal amphipathic helix of ATL2 (ATL2 Δ524–583), required for membrane fusion but not for membrane tethering, restored DENV replication in ATL2-KO cells to levels comparable with cells expressing WT ATL2 (Figs 5B and S5D) [41,92,100,101]. To confirm conservation between flaviviruses, these experiments were repeated with ZIKV. Similar to DENV-infected cells, only WT ATL2 and the Δ524–583 mutant showed significant rescue of ZIKV replication in ATL2-KO cells (Figs 5C, 5D, S5E, and S5F). Taken together, these results indicate that the primary biological function of ATL2 during flavivirus replication is membrane tethering, rather than the canonical membrane fusion typically associated with atlastin proteins.

Although the Δ524–583 ATL2 mutant has been shown to lack the capacity for membrane fusion in vitro, this deficiency has not been confirmed in living cells. To validate that rescue of virus replication in ATL2-KO cells expressing the Δ524–583 mutant was not due to residual membrane fusion activity of the mutant, we evaluated the effect of expressing Δ524–583 in ATL2-KO cells that were also depleted of ATL3. Since atlastin-1 is not expressed at measurable levels in these cells, depletion of both ATL2 and ATL3 significantly impairs ER branching, owing to the lack of atlastin-mediated ER fusion (Figs 5E and S5G) [38,101]. We therefore assessed whether transient expression of ATL2 Δ524–583 or WT ATL2 could restore ER morphology in cells lacking both ATL2 and ATL3. While expression of WT ATL2 successfully restored normal ER morphology, expression of ATL2 Δ524–583 did not (Figs 5E, 5F, and S5G–S5I). These results demonstrate that ATL2 Δ524–583, unlike WT ATL2, is not sufficient to restore ER fusion in the absence of other atlastin paralogs—confirming that this mutant is indeed fusion incompetent, and validating a role for ATL2-mediated membrane tethering in flavivirus replication.

Building on these results, we next investigated the ATL2 N-terminal hypervariable domain (HVD), which has been linked to membrane tethering. Specifically, recent reports suggest that the HVD of atlastin-1 facilitates the formation of homo-oligomeric structures that are proposed to build long membrane tethers [102]. We had previously shown that expression of an ATL2 N-terminal deletion mutant (ATL2 Δ2–65) did not result in the rescue of flavivirus replication in ATL2-KO cells [36]. To further investigate whether the ATL2 HVD functions to facilitate membrane tethering, we investigated the effect of overexpressing this domain on flavivirus infection, which, based on previous data, is predicted to compete for binding sites and disrupt tethering [102]. In cells overexpressing exogenous ATL2 HVD, we observed reduced DENV replication, approaching the level of reduction seen during ATL2 depletion (S5J and S5K Fig). These results confirm the importance of the ATL2 HVD in viral replication and further implicate ATL2-mediated membrane tethering as the critical function of ATL2 during infection.

### ATL2 function during flavivirus infection is not regulated by phosphorylation or alternative splicing

Atlastin-mediated membrane tethering and fusion have been primarily characterized using in vitro membrane fusion assays; as such, mechanisms of modulation in cells are still unclear. We therefore aimed to determine how ATL2 membrane tethering is regulated in flavivirus-infected cells. Based on our results indicating the importance of the ATL2 HVD in flavivirus infection, as well as previous studies showing hyperphosphorylation of the HVD, we hypothesized that HVD phosphorylation may regulate ATL2 membrane tethering function in flavivirus replication [92,102,103]. To test this, each serine and threonine residue in the ATL2 HVD was mutated to alanine to ablate potential phosphorylation sites (S6A Fig), and mutants were expressed in ATL2-KO or control cells, followed by infection with DV-R2A [104]. Surprisingly, expression of ATL2 phosphorylation mutants completely restored DENV replication in ATL2-KO cells, indicating that the ATL2 function relevant for flavivirus replication is not regulated by phosphorylation (S6B Fig). Expression of phosphorylation mutants did not lead to significant changes in cell viability in control or ATL2-KO cells (S6C Fig).

We next explored the role of alternative splicing in regulating ATL2 activity. There are two primary isoforms of ATL2, as well as three alternatively spliced isoforms which have been experimentally observed and seven additional isoforms that have been defined computationally [105,106]. To test if ATL2 isoforms are functional in restoring flavivirus replication in ATL2-KO cells (in which production of all ATL2 isoforms was ablated), five representative isoforms were expressed in ATL2-KO or control cells, followed by infection with DENV and evaluation of virus replication (S6D and S6E Fig). The only isoform that failed to restore viral replication was ATL2−3 (UniProt identifier Q8NHH9−3), which lacks the GTPase active site (S6D Fig) [106]. None of the isoforms tested negatively affected cell viability (S6F Fig). While we did not test all possible isoforms, the isoforms tested were representative of other predicted variants, indicating that alternative splicing of ATL2 likely does not have a significant impact on flavivirus replication.

### ATL2 activity can be targeted with a peptide to reduce flavivirus replication

**Fig 6:**
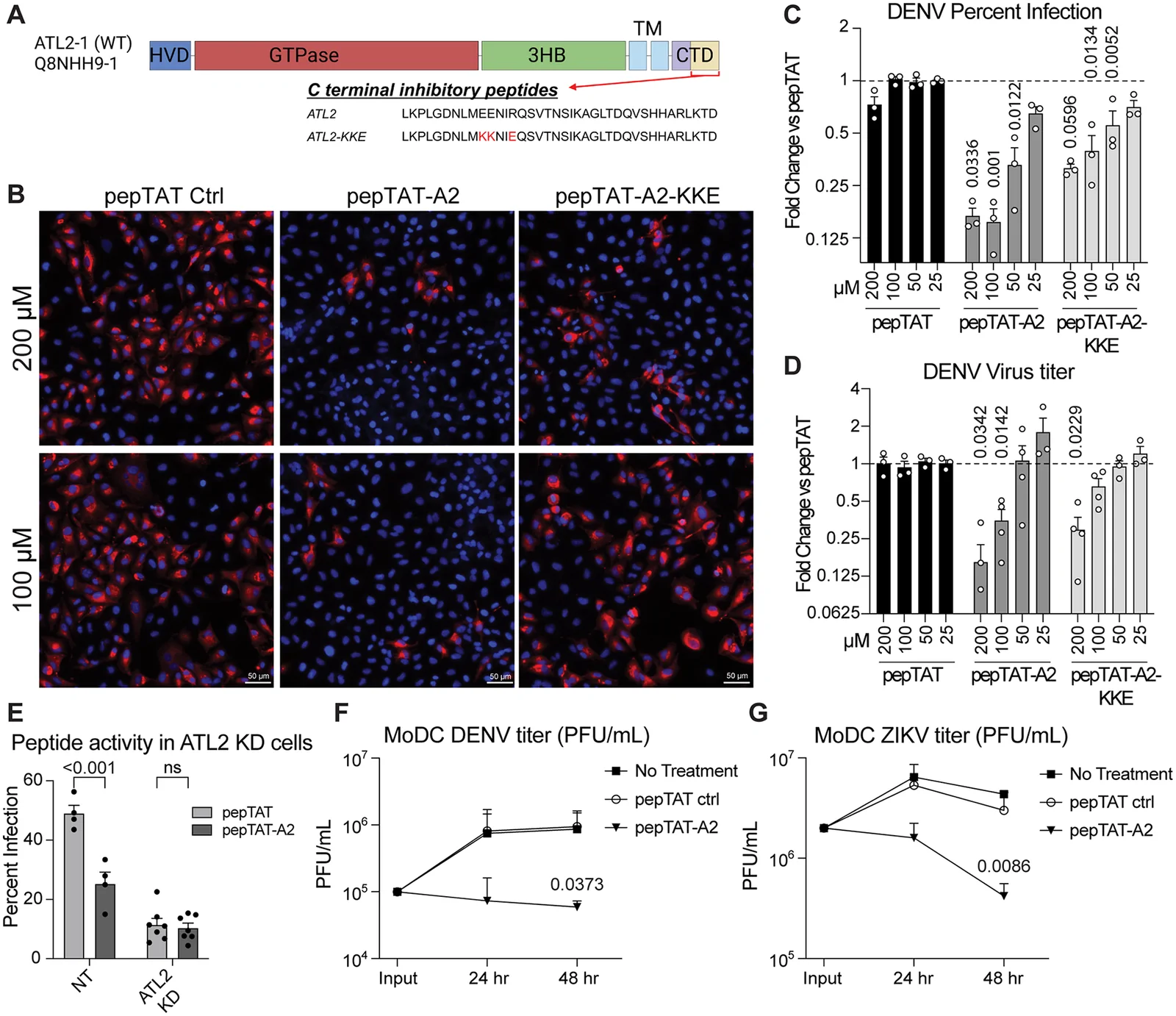
Peptides that phenocopy ATL2 inhibition limit flavivirus replication in immortalized and primary cells. **(A)** Schematic representation of the primary ATL2 isoform (ATL2−1, UniProt identifier Q8NHH9−1), highlighting the C-terminal autoinhibitory sequence. **(B–D)** A549 cells were treated with the indicated peptide at the indicated concentrations and infected with DENV at an MOI of 1 for 48 h. **(B)** Cells were fixed with PFA and viewed by immunofluorescence microscopy using an antibody specific for the viral NS3 protein (shown in red, with DAPI in blue). Images are representative of 4 independent experiments. **(C)** Graph shows the average fold change in percentage of infection for each peptide at the indicated concentration. **(D)** Graph shows the average fold change in virus titer from supernatants of cells treated with each peptide at the indicated concentration. **(E)** Cells were transduced with constructs encoding either NT shRNA or ATL2-targeted shRNA. After 48 h, cells were treated with 100 μM peptide as in **B–D** and infected with DENV for 48 h. Infection percentages were quantified as in **C**. Graph displays raw percent infection values per experiment; *n* ≥ 200 cells per condition. **(F, G)** moDC cells were treated with the indicated peptide at 100 µM and infected with either DENV **(F)** or ZIKV **(G)**. Supernatants were harvested at the indicated timepoints and virus was quantified by plaque assay. Graphs show the average PFU/mL at each time point. Graphs represent duplicate samples from 3 independent donors. For all graphs, statistical significance was determined using one-way ANOVA with Dunnett’s multiple comparison analysis. Significance shown for each sample is compared to pepTAT control samples at the same concentration or time point; error bars depict SEM. The data underlying this Figure can be found in S1 Data.

Having determined the significance of ATL2 in flavivirus replication, we next aimed to leverage this knowledge to inhibit viral replication. A previous report identified an autoinhibitory amino acid sequence within the ATL2 C-terminus that can be used as an exogenous peptide modulator of ATL2 function (Fig 6A), likely functioning in an inhibitory manner [103]. To test if this peptide could be used in cells to alter ATL2 activity and inhibit flavivirus replication, we added a cell-penetrating HIV-TAT sequence to this peptide (pepTAT-A2), applied it to A549 cells, infected these cells with DENV, and evaluated viral replication using immunofluorescence microscopy and plaque assay [107]. Strikingly, the percentage of virus-infected cells, as well as viral titers from infected cells, were significantly decreased upon treatment with pepTAT-A2 compared to cells treated with a pepTAT control peptide (Fig 6B–6D). To confirm the specificity of the peptide for ATL2, we also compared results to cells treated with a modified peptide containing three amino acid charge reversals (pepTAT-A2-KKE), which exhibits decreased interactions with ATL2 and approximately 50 percent less inhibitory activity in vitro [103]. Treatment with the pepTAT-A2-KKE peptide still led to moderate reductions in virus infection, but its antiviral activity was markedly attenuated (Fig 6B–6D). These experiments indicated that inhibition of ATL2 activity can significantly impair flavivirus infection.

Because these peptides were previously tested exclusively with in vitro assays, we performed several additional experiments to confirm specificity in cells [103]. To determine that the peptide is not acting directly upon virus particles in the supernatant, cells were treated with a peptide inhibitor without the cell-penetrating TAT sequence, which did not affect virus replication (S7A Fig). Additionally, FITC-conjugated versions of the pepTAT and pepTAT-A2 peptides were observed accumulating inside cells, indicating successful cell penetration (S7B Fig). In ATL3-KO cells (in which ATL2 is the predominant remaining atlastin paralog), pepTAT-A2 treatment of uninfected cells induced a marked morphological dysregulation of the ER (S7C and S7D Fig). Furthermore, activity of pepTAT-A2 was abrogated in ATL2 KD cells (Fig 6E). Finally, none of the peptide treatments had a significant impact on cell viability in control cells (S7E Fig), but significant impairment of viability was noted in pepTAT-A2-treated cells depleted of ATL3, in which ATL2 is the primary remaining atlastin (S7F Fig). This impact was comparable to cells depleted of both ATL2 and ATL3 by shRNA-mediated knockdown (S7G Fig). These results suggest that treatment with this peptide correlates with an inhibition of ATL2 function, and thus the effect of pepTAT-A2 on viral replication is likely specific to its interaction with ATL2.

To confirm our observations in a more biologically relevant system, we evaluated the effect of peptide treatment in primary human monocyte-derived dendritic cells (moDCs). These cells are among the first sites of viral replication upon natural infection and are important for establishing and spreading flavivirus infections within human hosts [108–110]. moDCs differentiated from human peripheral blood mononuclear cells (PBMCs) were incubated with the indicated peptides and infected with either DENV or ZIKV [74–76]. Productive infection in moDCs was confirmed by verifying a significant loss of viral titers upon addition of a pan-flavivirus polymerase inhibitor (NITD008) (S7H and S7I Fig) [111,112]. Paralleling results from A549 cells, treatment with pepTAT-A2 significantly reduced titers and intracellular viral RNA levels for both viruses, as compared to the pepTAT control peptide, without impacting cell viability (Figs 6F, 6G, and S7H–S7J). Thus, across both immortalized and primary human cells, our results demonstrate that ATL2 function can be targeted to inhibit flavivirus infection.

## Discussion

**Fig 7:**
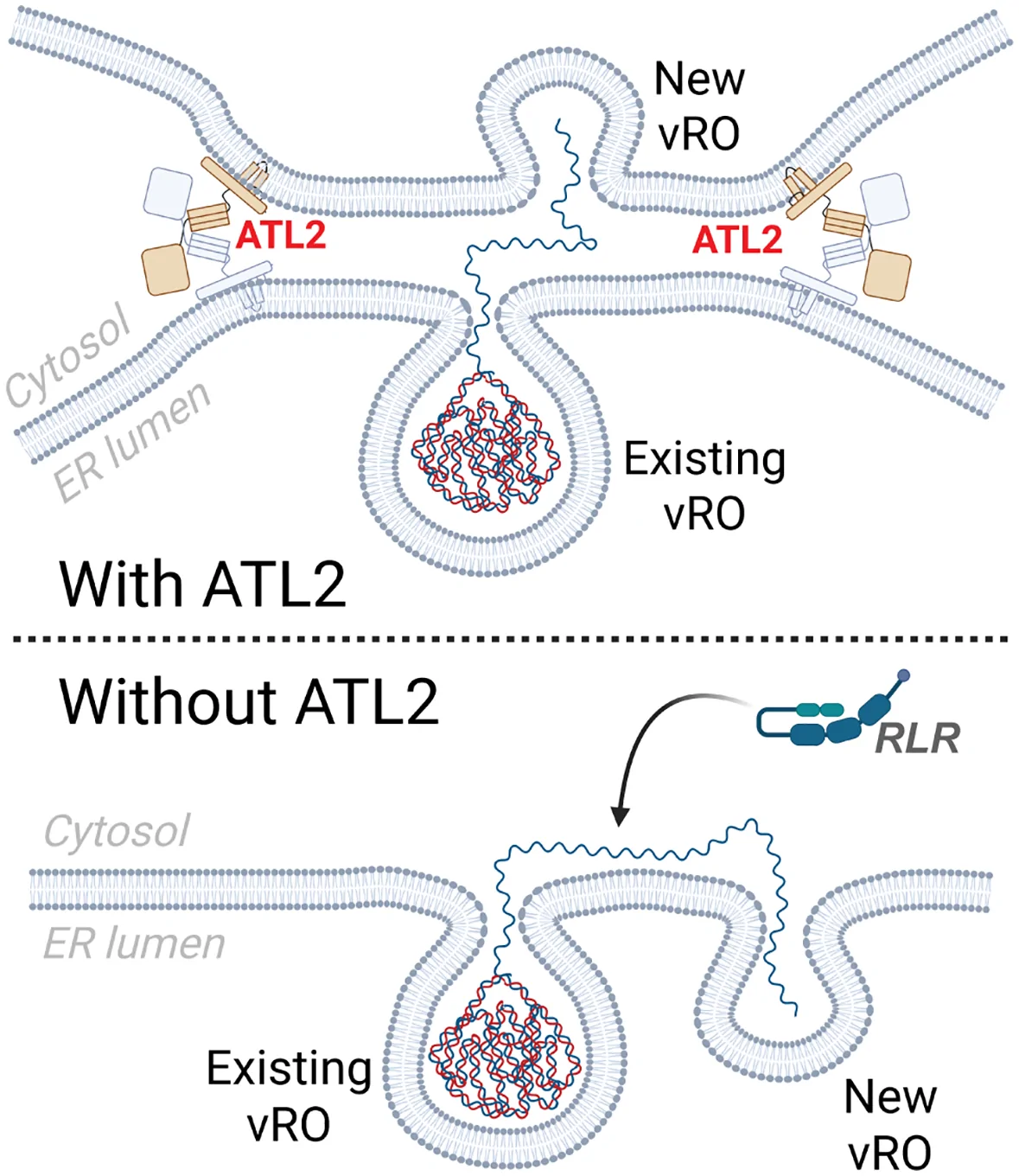
Model: ATL2 membrane tethering is critical for the spatial organization of flavivirus genome replication. ATL2 tethering dimers bring ER membranes into close contact, facilitating the trafficking of newly synthesized viral genomes from existing vROs into vROs forming on juxtaposed membranes (top). This close connection forms a microdomain which brings together the necessary factors for nascent vRO formation and shields the cytosol-exposed viral RNA from innate immune sensors. Because membrane tethering is reversible, this also permits the expansion of viral genome replication away from sites of existing vRO biogenesis. In the absence of ATL2 membrane tethering (bottom), viral genomes extruded from the vRO are exposed to the cytosol and more likely to form new vROs on the same membrane, leading to the concentration of sites of vRO biogenesis and increased recognition by RIG-I-like receptors (RLRs) in the cytosol. Citation to Use: Created in BioRender. Neufeldt, C. (2026), https://app.biorender.com/citation/6a0334e4293894be54bd3d35.

Defining the membrane reorganization mechanisms that govern vRO biogenesis and RNA trafficking in infected cells is critical to understanding flavivirus infection. Here, we present evidence that the spatial organization of flavivirus genome replication is mediated by the membrane tethering capability of the host factor ATL2. Depletion of ATL2 in flavivirus-infected cells impeded proper vRO formation and reduced virus replication, which correlated with a robust increase in cellular immune activation. While the membrane tethering activity of ATL2 was required for productive flavivirus infection, its membrane fusion activity proved dispensable. Based on these findings, we propose a model in which the organization and distribution of flavivirus vROs in the cytoplasm of infected cells is mediated by homotypic ER membrane tethers (Fig 7). This organization allows newly synthesized viral genomes egressing from existing vROs to form de novo vROs on juxtaposed ER membranes. The reversible quality of ER tethers then allows the dispersion of vROs throughout the cell as the ER is dynamically rearranged [41,92,96,97,113,114]. Without ER membrane tethers, viral RNA remains confined to *cis*-ER membranes, resulting in the accumulation of vROs and viral RNA at the initial site of replication. Our data also suggest that ER membrane tethers may have an immune-protective role, potentially limiting access of pattern recognition receptors (PRRs) to viral RNA intermediates. Together, these findings indicate that ER-to-ER contacts are needed to assemble specific membrane microdomains that are critical for sustaining flavivirus replication.

Our evaluation of DENV-infected cells by electron tomography (ET) revealed numerous membrane contact sites between vROs and juxtaposed membranes. These results parallel previous findings for DENV and other flaviviruses, including recent observations for tick-borne encephalitis virus (TBEV) using in situ cryo-ET [18,19,27]. In both DENV- and TBEV-infected cells, smaller vROs were often found along ER membranes tightly juxtaposed to other ER membranes, potentially representing intermediates of vRO formation before genome replication is established. These observations suggest that membrane contacts also contribute to proper vRO maturation. This is supported by our data showing decreased genome replication and enrichment of smaller vROs in ATL2-depleted cells. Furthermore, ET has also demonstrated the tight membrane contacts that occur at the viral assembly site, where the juxtaposition of ER membranes enables direct transfer of genomic RNA from the vRO into a budding virion [18–20,27,115]. This arrangement likely improves the efficiency of virus production by concentrating required viral assembly components in one location. In ATL2-depleted cells, we observed very few virions by electron microscopy and, indeed, significantly decreased viral titers in supernatants, suggesting that ATL2 may also be required to establish the membrane contacts needed for virion assembly. Taken together with prior studies, our results support the importance of ER-to-ER membrane contacts for vRO maturation and perhaps for virion budding.

In addition to reducing viral replication, ATL2 depletion and the resulting disruption of ER membrane tethers increased cellular immune activation. A key predicted function of vROs is to sequester replication machinery from the cytosol, thereby shielding replication intermediates from immune receptors such as RIG-I and MDA5 [17,21,22]. Increased immune activation in ATL2-depleted, flavivirus-infected cells suggests that these receptors have greater access to viral genomic material. This conclusion is corroborated by the observation that, as compared to control cells, a greater proportion of infected ATL2-depleted cells exhibited dsRNA signal when permeabilized with digitonin prior to immunostaining. These results indicate that more dsRNA may accumulate in the cytosol in ATL2-depleted cells, potentially due to aberrant release from vROs or structural deficiencies which incompletely sequester dsRNA, leading to increased innate immune activation. It is also possible that increased exposure of single-stranded (ssRNA) moieties contributes to increased immune recognition. Since vROs still assemble without ATL2, immune activation may occur due to trafficking disruptions once newly synthesized ssRNA exits the vRO—particularly given that RIG-I can recognize both dsRNA and 5′-triphosphate ends on ssRNA [116]. Our data suggest that membrane connections may create a cellular microdomain that restricts PRR access, allowing viral RNA to traffic between membranes undetected. Alternatively, changes in vRO morphology and distribution in ATL2-depleted cells may reduce capping efficiency, leading to more 5′-triphosphate ends detectable by RIG-I.

We also noted that in ATL2-depleted cells, there was a slight enrichment for inflammatory and TNF gene upregulation, which could impact immune activation and flavivirus infection. However, previous studies have demonstrated that although TNF and inflammation play a role in DENV disease severity in patients, treatment with TNF does not directly impact viral replication [117–119]. Additionally, we show that ATL2 depletion does not impact immune activation or viral replication in alphavirus-infected cells, indicating that the immune activation and reduction in viral replication observed in flavivirus infection are specific and not a result of general inflammatory changes from ATL2 depletion. Clarifying the mechanisms of immune activation in the absence of ATL2 will be the focus of future studies.

Our data show that ER membrane tethering has a biological role during infection, which may extend to other aspects of cellular homeostasis. Atlastin proteins are best known for mediating ER tubule fusion, yet the low intrinsic fusion activity of the predominant ATL2 isoform and in vitro evidence of reversible, ATL2-mediated liposome tethering support a role for ATL2 membrane tethering in regulating cellular functions. However, to our knowledge, only one other study has shown a biological relevance for atlastin tethering, specifically showing that the membrane tethering function of atlastin-1 facilitates COPII-coated vesicle trafficking in neurons [40]. These conclusions were predicated upon the use of a fusion mutant which has been shown to also display tethering deficiencies, and this work has not yet been replicated for ATL2 [41,92,99]. Building on our results implicating ATL2 in viral RNA trafficking, we speculate that ATL2 may also mediate trafficking of cellular factors. Since transcriptomic and proteomic analyses of ATL2-depleted cells did not reveal broad translational changes, it is less likely that mRNA trafficking is altered; however, it is possible that specific protein or lipid distributions are altered. Alternatively, ATL2-mediated tethers may have other functions in maintaining ER structure and homeostasis. Understanding how ATL2 fusion and tethering are regulated will be key to determining how these activities contribute to cellular functions.

Combined with our previous work, the data shown here demonstrate that ATL2 and ATL3 have different functions during flavivirus replication, with ATL3 influencing virion maturation and ATL2 functioning in vRO organization [36]. Recent studies examining atlastin protein fusion and tethering activities have identified that N- and C-terminal elements of these proteins differentially regulate atlastin function [43,92,102,103,120,121]. These regions differ significantly between atlastin paralogs; indeed, recent work has shown that due to the absence of an autoinhibitory C-terminal element, ATL3 possesses greater fusogenic activity than ATL2 [121]. It is therefore tantalizing to speculate that ATL2 is preferentially utilized for vRO biogenesis during flavivirus infection due to its decreased membrane fusion activity. It is also possible that the localization of ATL2 and ATL3 to different microdomains of the ER impacts their role in flavivirus infection. Our findings also support a growing body of evidence that, in addition to redundant functions in membrane fusion, ATL2 and ATL3 also have paralog-specific activities. Regardless, here we demonstrate that ATL2 has specific functions in vRO biogenesis and that providing excess amounts of either the ATL2 N-terminal HVD or the ATL2-1 C-terminal autoregulatory sequence specifically modulates ATL2 activity and reduces viral replication in both immortalized and primary cells. The impact of ATL2 modulation upon flavivirus replication and specifically vRO formation highlights intriguing avenues for future research.

In this study, we used the autoinhibitory C-terminal ATL2 sequence mentioned above as an exogenous peptide inhibitor of flavivirus replication. Our data suggest that treatment with this peptide most likely limits flavivirus replication by the inhibition of ATL2 itself, demonstrating a proof-of-principle that targeting host factor activity can reduce viral replication. However, it is important to note that in vitro, this peptide has only been shown to limit the function of an ATL2 mutant that already lacks the C-terminal autoregulatory domain; therefore, the peptide has only been directly demonstrated to inhibit ATL2 that has been relieved of autoinhibition [103]. Because the mechanism for activation of endogenous ATL2 that still possesses its C-terminus is unknown, the further inhibitory activity of the peptide has not yet been tested in vitro. Nevertheless, our results show that the detrimental impact of peptide treatment on flavivirus replication is relieved in the absence of ATL2. In addition to several controls that demonstrate the peptide does not exhibit off-target effects, these results strongly implicate inhibition of ATL2 function as the primary mechanism by which this peptide interferes with flavivirus replication.

Conservation of vRO morphology among flaviviruses makes defining the structure and function of vROs highly relevant for both understanding fundamental flavivirus biology and identifying potential therapeutic targets. Indeed, several recent studies have illustrated the antiviral potential of drugs which disrupt vRO assembly and maintenance [122–125]. However, little is known about the mechanisms of vRO biogenesis and, in particular, the importance of host factors in the formation and function of these complexes. Our data provide evidence that a mechanistic understanding of host factor function during flavivirus infection can inform target selection for antiviral development. Defining how host factors coordinate vRO formation could expand the potential for host-targeted antivirals to treat flavivirus infections. Given the essential function of vROs in orchestrating flavivirus replication, such interventions possess significant potential for broad-spectrum activity [10,11].

In summary, we show that ATL2-mediated membrane tethering serves as a critical link in the chain of events that facilitate a productive flavivirus infection. The role of ATL2 tethering in facilitating infection is conserved for multiple flaviviruses, representing a common host mechanism in flavivirus replication. This knowledge reinforces the importance of spatial coordination of viral processes during infection, sheds new light upon molecular mechanisms of ER regulation, and illuminates a specific and conserved relationship between flaviviruses and their hosts.

## Supporting information

S1 Fig ATL2 depletion decreases flavivirus replication and alters vRO distribution. A549 cells were transduced with constructs encoding for shRNA directed against ATL2 (ATL2 KD), or a nontargeting shRNA (NT). **(A and B)** Ninety-six hours after transduction, ATL2 mRNA levels were determined by RT-qPCR, and protein levels were determined by western blot. **(A)** Graph shows the average relative level of ATL2 mRNA in transduced cells. **(B)** Figure displays western blot stained for ATL2 (top) and GAPDH (bottom). The upper band in the top blot (indicated by a black arrow) is ATL2; the lower band is nonspecific. **(C)** Transduced cells were infected with DENV serotype 1 (WP75 – DV1), serotype 2 (16681 – DV2), serotype 3 (H87 – DV3) or serotype 4 (H241 – DV4) for 48 h, followed by evaluation of virus titer by FFU assay. Graphs show the mean fold change in FFU/mL relative to the NT condition. **(D)** Ninety-six hours after transduction, cell viability was evaluated by CellTiter-Blue assay in uninfected cells. Graph shows the average fold change in cell viability, as compared to the NT condition. **(E–J)** Forty-eight hours after transduction with ATL2 shRNA or NT shRNA constructs, cells were infected with DENV (panels **E–G**) or ZIKV (panels **H–J**) for 24 h, followed by fixation and processing for viewing by TEM. Centrographic statistics were used to quantify vRO distributions. Graphs show the average nearest neighbor distance per cell (**E** and **H**), the average second-nearest neighbor distance per cell (**F** and **I**), and the standard distance of each cell’s vRO distribution (**G** and **J**) for each virus; *n* ≥ 8 cells per condition. We note that for **H** and **J**, the presence of a clear outlier reduced statistical power. For all graphs, statistical significance was determined using Welch’s *t* test, and error bars depict standard error of the mean (SEM). The data underlying this Figure can be found in S1 Data.
(TIF)

S2 Fig ATL2 depletion does not impact cellular or viral translation. **(A–E)** Cells were transduced with ATL2-specific or NT shRNA-expressing constructs for 96 h, followed by total RNA or protein extraction for RNA-seq or quantitative mass spectrometry analysis, respectively (*n* = 4 biological replicates). **(A)** Transcriptomic analysis of ATL2 KD cells. Volcano plot showing differentially expressed genes in ATL2 KD vs. NT A549 cells in bulk RNA-seq. Significantly up- or down-regulated genes are displayed in red or blue, respectively (*p*-adj < 0.1 and |log2-fold-change| > 1). Labels indicate cellular transcripts displaying a |log2-fold-change| > 2. **(B)** Proteomic analysis of ATL2 KD cells. Volcano plot showing differentially expressed proteins in ATL2 KD vs. NT A549 cells in global proteomic analysis by LC–MS/MS. Significantly up- or down-regulated proteins are displayed in red or blue, respectively (Student *t* test *p*-value < 0.05 and |log2-fold-change| > 2). **(C)** Intersection of transcriptomic and proteomic analysis of ATL2 KD cells. Scatter plot displays differential expression of transcripts and proteins in ATL2 KD cells. Labels indicate cellular proteins up- or down-regulated at the protein level (|log2-fold-change| > 2), which display a milder or absent change at the mRNA level (0 > |log2-fold-change| > 1). **(D and E)** Plots show the gene set enrichment analysis employing the MSigDB collection of Hallmark pathways (FDR cutoff of 0.001) for ATL2 KD cells compared to NT cells. **(D)** Displays enriched pathways from genes where transcripts increased in ATL2 KD cells. **(E)** Displays enriched pathways from genes where transcripts decreased in ATL2 KD cells. **(F)** Schematic representation of the pIRO expression cassette. **(G and H)** Lunet-T7 cells were transduced with ATL2 or NT shRNA for 48 h, followed by transfection with either pIRO-D or pIRO-Z constructs. Eighteen hours later for pIRO-D **(G)**, or 16 h later for pIRO-Z **(H)**, cells were fixed, stained for viral NS3 and imaged by epifluorescence microscopy. Images are representative of NS3 staining. Scale bar, 20 µm. Graphs show the average percentage of transfected cells for 3 independent experiments; statistical significance was determined using Welch’s *t* test, and error bars represent SEM. ns = not significant. The data underlying this Figure can be found in S1 Data.
(TIF)

S3 Fig ATL2 KD alters immune activation in flavivirus, but not alphavirus-infected cells, and reduced flavivirus replication during ATL2 KD can be partially rescued by concomitant knockdown of MAVS. **(A–F)** Cells were transduced with constructs encoding for ATL2-directed shRNA or NT shRNA. **(A)** Ninety-six hours after transduction, cells were lysed and ATL2 mRNA levels were determined by qPCR. Graph shows the average fold change in ATL2 transcript levels. **(B–F)** Forty-eight hours after transduction, cells were infected with the indicated virus or mock-infected for 24 h, followed by evaluating mRNA levels of the indicated genes by RT-qPCR. **(B)** Graph shows the average fold change in flavivirus RNA between ATL2 KD and NT cells. **(C–F)** Graphs show the mean fold change in the indicated transcript level in alphavirus-infected cells, as compared to mock-infected NT cells. **(G–K)** Cells were simultaneously transduced with constructs encoding ATL2 shRNA and MAVS shRNA, each at MOI 5. Forty-eight hours after transduction, cells were infected with the indicated flavivirus for 48 h, followed by evaluation of RNA levels by RT-qPCR. **(G and H)** Graphs show the average fold change in viral genome content in comparison to NT cells. **(I and J)** Graphs show the average fold change in ATL2 **(I)** or MAVS **(J)** intracellular mRNA levels, as compared to the NT condition. **(K)** Ninety-six hours after transduction, cell viability was evaluated by CellTiter-Blue assay in uninfected cells. Graph shows the average fold change in cell viability, as compared to the NT condition. The dotted line indicates an 80% threshold below which viability would be considered impaired. For all graphs, statistical significance was determined using Welch’s *t* test; the results of each pairwise *t* test are displayed simultaneously. Error bars show SEM and ns = not significant. The data underlying this Figure can be found in S1 Data.
(TIF)

S4 Fig Colocalization of ER proteins with sites of flavivirus replication. **(A)** A549 cells expressing HA-tagged ATL2 were infected with DENV for 24 h, then fixed and stained for NS3, dsRNA, and HA using specific antibodies. Panels show representative images for mock-infected and DENV-infected cells. Scale bar = 5 µm. **(B)** Graph shows the average Pearson’s correlation coefficients for the indicated fluorescence signals for 12 images (*n* ≥ 30 cells per condition). Error bars depict SEM. **(C)** A549 cells were infected with ZIKV for 24 h, then fixed and stained for reticulon-3 (RTN3) and dsRNA. Panels show representative images for mock-infected and ZIKV-infected cells. Scale bar = 5 µm. **(D)** 24 h after infection with DENV or ZIKV, cells expressing untagged ATL2 were fixed and subjected to proximity ligation assays (PLA) using anti-HA and anti-NS5 antibodies. Cells were also immunostained for the viral NS3 protein to identify infected cells. Cells were imaged using confocal microscopy. Scale bar = 20 µm. The data underlying this Figure can be found in S1 Data.
(TIF)

S5 Fig Expression of a membrane tethering-competent ATL2 mutant restores flavivirus replication in ATL2-KO cells. **(A)** ATL2-KO and control single-cell clones were isolated from knockout and control populations and evaluated for ATL2 presence by Western blotting, then back-pooled. Columns 1–5, control (“Ctrl”) single-cell clones. Column 6, back-pooled control cells. Columns 7–11, ATL2-KO (“KO”) single-cell clones. Column 12, back-pooled ATL2-KO cells. **(B–F)** Ctrl and ATL2-KO cells were transduced with ATL2 mutants for 24 h, followed by infection with the indicated flaviviruses for 48 h. **(B)** Expression of all mutants was verified using western blot. **(C)** Graph shows relative cell viability of uninfected cells 72 h post-transduction, as evaluated by CellTiter Blue assay. Results are normalized to ctrl cells transduced with an empty vector (leftmost bar). **(D–F)** ATL2 mutants or empty lentiviral vectors were transduced into control (ctrl) and ATL2 knockout cells (ATL2 KO). **(D)** Graph shows the average fold change in *Renilla* luciferase (RLuc) signal from DV R2A replication in cells expressing the indicated ATL2 mutants. **(E)** Graph shows the average fold change in ZIKV RNA levels in cells expressing the indicated ATL2 mutants (measured by qPCR). **(F)** Graph shows the average fold change in ZIKV titers (PFU/mL) harvested from cells expressing the indicated ATL2 mutants (measured by plaque assay). For graphs in **(D–F)**, results were normalized to viral replication in ctrl cells expressing an empty lentiviral vector (left bar in each graph). **(G and H)** ATL2 KO cells were transduced with lentivirus constructs expressing ATL3-targeted shRNA for 48 h, then transduced with constructs expressing either ATL2 Δ524–583 or WT ATL2. Forty-eight hours later, ER morphology was assessed by immunostaining for reticulon-3 (RTN3). **(G)** Representative images display RTN3 signal in gray and DAPI in blue. For comparison, control cells and ATL2-KO/ATL3 KD cells transduced with empty vectors are included at left. Scale bar = 5 µm. **(H)** Manual quantification of cells with properly reticulated ER was performed; graph shows the proportion of cells in each condition which displayed normal ER morphology. *n* ≥ 38 cells and 3 images per condition. **(I)** Graph shows ATL3 levels relative to ctrl cells for experiments shown in **G and H**, measured by qPCR. Statistical significance was determined using one-way ANOVA with Dunnett’s multiple comparison analysis. **(J and K)** Cells stably overexpressing the ATL2 N-terminal hypervariable domain (HVD—amino acids 1–65) or WT cells were transduced with ATL2 or NT shRNA constructs for 48 h. Cells were then infected with DENV for 48 h. Graphs show the average fold change in intracellular viral RNA **(J)** or viral titers in supernatants from infected cells **(K)** compared to NT cells. For all graphs except **I**, statistical significance was determined by two-way ANOVA with Tukey’s HSD. Error bars for all graphs depict SEM; ns = not significant. The data underlying this Figure can be found in S1 Data.
(TIF)

S6 Fig ATL2 function during flavivirus infection is not regulated by phosphorylation or alternative splicing. **(A)** AlphaFold2 ribbon model of ATL2. ATL2 domains: Dark blue—HVD, red—GTP-binding domain (GTPase), green—3-helix bundle (3HB), light blue—transmembrane helices (TM), purple—C-terminal amphipathic helix, tan—C-terminal domain (CTD). Residues mutated in **B** are highlighted in light orange (S24 and S27), dark orange (all other HVD serines), or maroon (HVD threonines). **(B and C)** ATL2 phospho-mutants or empty lentiviral vectors were transduced into control and ATL2-KO cells. After 24 h, cells were infected with DV-R2A and monitored for viral replication using luciferase expression. **(B)** Graph shows the average fold change in RLuc signal from DENV replication compared to control cells transduced with the empty vector. **(C)** Graph shows the relative change in the viability of uninfected cells compared to control cells transduced with an empty vector. **(D)** Schematic of the gene sequences of the ATL2 isoforms used in **E** and **F**. Domains colored similarly to **A**. **(E and F)** ATL2 isoforms (indicated by their UniProt code) or empty lentiviral vectors were transduced into control and ATL2-KO cells. After 24 h, cells were infected with DV-R2A and monitored for viral replication using luciferase expression. **(E)** Graphs show the average fold change in RLuc signal compared to control cells transduced with the empty vector. **(F)** Graph shows cell viability at 72 h post-transduction in uninfected cells (normalized to control cells transduced with the empty vector). The dotted line indicates an 80% threshold below which viability would be considered impaired. For all graphs, statistical significance was determined by two-way ANOVA with Tukey’s HSD, and error bars show SEM. The data underlying this Figure can be found in S1 Data.
(TIF)

S7 Fig A peptide that phenocopies ATL2 inhibition restricts flavivirus infection. **(A)** A549 cells were treated with ATL2 peptides lacking the TAT cell-penetrating sequence, or a control peptide containing only the TAT sequence (pepTAT), at 100 µM and infected with DENV (MOI 1) for 48 h. Graph shows the average fold change in percentage of infection (left bars) and the average fold change in virus titer (right bars) for each peptide. **(B)** A549 cells were treated with FITC-conjugated peptides for 16 h, then imaged using confocal microscopy. Representative images are shown. Scale bar = 20 µm. **(C and D)** A549 cells were transduced with constructs encoding NT or ATL3-targeted shRNA. After 48 h, cells were treated with the indicated peptides (100 µM) for 16 h, then immunostained for PDI (green) and DAPI (blue), followed by imaging using confocal microscopy (Scale bar = 10 µm). Representative images are shown in **C**; insets at far right are magnifications of indicated boxed areas. Manual quantification of the percentage of cells with normal ER morphology is shown by the graph in **D**; *n* ≥ 80 cells per condition. **(E)** A549 cells were treated with the indicated peptide at the indicated concentration for 48 h and cell viability was evaluated by CellTiter-Blue assay. Graph shows the average fold change in cell viability compared to pepTAT-treated cells. **(F)** A549 cells were transduced with nontargeting shRNA constructs (NT) or constructs targeting ATL2 or ATL3. Forty-eight hours later, cells were treated with the indicated peptide for 24 h and cell viability was assessed by CellTiter-Blue assay. Results are normalized to NT cells treated with pepTAT-A2. **(G)** A549 cells were transduced with nontargeting shRNA constructs (NT) or constructs targeting ATL2 and/or ATL3. After 24 h, viability was assessed by CellTiter-Blue assay. Results are normalized to NT cells. **(H–J)** moDC were treated with the indicated peptide (100 µM) or NITD-008 inhibitor (10 µM) and infected with either DENV, ZIKV, or mock-infected for 48 h. Graphs represent duplicate samples from 3 independent donors. **(H and I)** Graphs show the average fold change in intracellular virus RNA for each virus. **(J)** Graph shows the average fold change in cell viability for mock-infected cells, normalized to pepTAT-treated cells. Dotted line indicates an 80% threshold below which viability would be considered impaired. For **(F–I)**, Statistical significance was determined using one-way ANOVA with Dunnett’s multiple comparison analysis. Significance shown for each sample is compared to pepTAT control samples. For all graphs, error bars show SEM. The data underlying this Figure can be found in S1 Data.
(TIF)

S1 Table Cloning primers. List of cloning primers used in this study.
(DOCX)

S2 Table RT-qPCR primers. List of RT-qPCR primers used in this study.
(DOCX)

S3 Table Antibodies. List of Antibodies used in this study.
(DOCX)

S4 Table Peptide sequences. Sequences for peptides used in this study.
(DOCX)

S1 Data Source data underlying the quantitative results presented in the manuscript. Excel workbook containing the individual numerical values underlying the graphs and statistical analyses in the main and supplementary figures.
(XLSX)

S1 Raw Images Uncropped Western blots corresponding to the immunoblot data presented in the manuscript. Uncropped images of the original Western blots used to generate the cropped panels shown in the main and supplementary figures. The corresponding figure and panel are indicated for each blot. Molecular weight markers are indicated.
(PDF)

## Abbreviations

AGC: automatic gain control
ATL2: atlastin-2
BSA: bovine serum albumin
CHIKV: chikungunya virus
CMC: carboxymethylcellulose
Ct: cycle threshold
DDA: data-dependent acquisition mode
DENV: dengue virus
DMEM: Dulbecco’s modification of Eagle’s medium
EM: electron microscopy
ER: endoplasmic reticulum
ET: electron tomography
FA: formic acid
FFU: focus-forming unit
GM-CSF: Granulocyte-macrophage colony-stimulating factor
HCD: higher-energy collisional dissociation
HPRT: hypoxanthine phosphoribosyltransferase 1
HSD: Honestly Significant Difference
HVD: hypervariable domain
iBAQ: intensity-based absolute quantification
IF: immunofluorescence
IL-4: interleukin-4
IRB: Institutional Review Board
ISGs: interferon-stimulated genes
MAVS: mitochondrial antiviral signaling protein
MEM: minimum essential medium
moDCs: monocyte-derived dendritic cells
NT: nontarget
ONNV: O’nyong’nyong virus
PBMC: peripheral blood mononuclear cell
PFU: plaque-forming unit
pIRO: plasmid-induced replication organelle
PLA: proximity ligation assays
POWV: Powassan virus
PRRs: pattern recognition receptors
RNA-seq: RNA sequencing
RT-qPCR: reverse transcription-quantitative PCR
SDS: sodium dodecyl sulphate
SEM: standard error of the mean
SG-PERT: SYBR Green I-based real-time PCR-enhanced reverse transcriptase
shRNA: short hairpin RNA
TBEV: tick-borne encephalitis virus
TEM: transmission electron microscopy
vROs: viral replication organelles
WNV: West Nile virus
WT: wildtype
YFV: yellow fever virus
ZIKV: Zika virus

## References

[1] Messina JP, Brady OJ, Golding N, Kraemer MUG, Wint GRW, Ray SE, et al. The current and future global distribution and population at risk of dengue. Nat Microbiol. 2019;4(9):1508–15. doi: 10.1038/s41564-019-0476-8 31182801 PMC6784886

[2] Sanjuán R, Nebot MR, Chirico N, Mansky LM, Belshaw R. Viral mutation rates. J Virol. 2010;84:9733–48. 20660197 10.1128/JVI.00694-10 PMC2937809

[3] Pierson TC, Diamond MS. The continued threat of emerging flaviviruses. Nat Microbiol. 2020;5(6):796–812. doi: 10.1038/s41564-020-0714-0 32367055 PMC7696730

[4] Liang Y, Dai X. The global incidence and trends of three common flavivirus infections (Dengue, yellow fever, and Zika) from 2011 to 2021. Front Microbiol. 2024;15:1458166. 39206366 10.3389/fmicb.2024.1458166 PMC11349664

[5] Chancey C, Grinev A, Volkova E, Rios M. The global ecology and epidemiology of West Nile virus. Biomed Res Int. 2015;2015:376230. doi: 10.1155/2015/376230 25866777 PMC4383390

[6] IHME Pathogen Core Group. Global burden associated with 85 pathogens in 2019: a systematic analysis for the Global Burden of Disease Study 2019. Lancet Infect Dis. 2024;24(8):868–95. doi: 10.1016/S1473-3099(24)00158-0 38640940 PMC11269650

[7] Piantadosi A, Solomon IH. Powassan virus encephalitis. Infect Dis Clin North Am. 2022;36(3):671–88. doi: 10.1016/j.idc.2022.03.003 36116842 PMC9494578

[8] Rabe IB, Hills SL, Haussig JM, Walker AT, Dos Santos T, San Martin JL, et al. A review of the recent epidemiology of Zika virus infection. Am J Trop Med Hyg. 2025;112(5):1026–35. doi: 10.4269/ajtmh.24-0420 39933180 PMC12062665

[9] Bifani AM, Chan KWK, Borrenberghs D, Tan MJA, Phoo WW, Watanabe S, et al. Therapeutics for flaviviral infections. Antiviral Res. 2023;210:105517. doi: 10.1016/j.antiviral.2022.105517 36592668

[10] Cakir M, Obernier K, Forget A, Krogan NJ. Target discovery for host-directed antiviral therapies: application of proteomics approaches. mSystems. 2021;6:e00388-21. 10.1128/mSystems.00388-21 PMC8547474 34519533

[11] Mahajan S, Choudhary S, Kumar P, Tomar S. Antiviral strategies targeting host factors and mechanisms obliging +ssRNA viral pathogens. Bioorg Med Chem. 2021;46:116356. doi: 10.1016/j.bmc.2021.116356 34416512 PMC8349405

[12] Karim M, Mishra M, Lo C-W, Saul S, Cagirici HB, Gourdelier M, et al. PIP4K2C inhibition reverses autophagic flux impairment induced by SARS-CoV-2. Nat Commun. 2025;16(1):6397. doi: 10.1038/s41467-025-61759-1 40640184 PMC12246422

[13] Neufeldt CJ, Cortese M, Acosta EG, Bartenschlager R. Rewiring cellular networks by members of the Flaviviridae family. Nat Rev Microbiol. 2018;16(3):125–42. doi: 10.1038/nrmicro.2017.170 29430005 PMC7097628

[14] Gillespie LK, Hoenen A, Morgan G, Mackenzie JM. The endoplasmic reticulum provides the membrane platform for biogenesis of the flavivirus replication complex. J Virol. 2010;84(20):10438–47. doi: 10.1128/JVI.00986-10 20686019 PMC2950591

[15] Nicholls CMR, Sevvana M, Kuhn RJ. Structure-guided paradigm shifts in flavivirus assembly and maturation mechanisms. Adv Virus Res. 2020;108:33–83. doi: 10.1016/bs.aivir.2020.08.003 33837721 PMC7510438

[16] Neufeldt CJ, Cortese M. Membrane architects: how positive-strand RNA viruses restructure the cell. J Gen Virol. 2022;103(8):10.1099/jgv.0.001773. doi: 10.1099/jgv.0.001773 35976091

[17] Överby AK, Popov VL, Niedrig M, Weber F. Tick-borne encephalitis virus delays interferon induction and hides its double-stranded RNA in intracellular membrane vesicles. J Virol. 2010;84:8470–83. 20554782 10.1128/JVI.00176-10 PMC2919015

[18] Welsch S, Miller S, Romero-Brey I, Merz A, Bleck CKE, Walther P, et al. Composition and three-dimensional architecture of the dengue virus replication and assembly sites. Cell Host Microbe. 2009;5(4):365–75. doi: 10.1016/j.chom.2009.03.007 19380115 PMC7103389

[19] Cortese M, et al. Ultrastructural characterization of Zika virus replication factories. Cell Reports. 2017;18:2113–23. 28249158 10.1016/j.celrep.2017.02.014 PMC5340982

[20] Junjhon J, Pennington JG, Edwards TJ, Perera R, Lanman J, Kuhn RJ. Ultrastructural characterization and three-dimensional architecture of replication sites in dengue virus-infected mosquito cells. J Virol. 2014;88(9):4687–97. doi: 10.1128/JVI.00118-14 24522909 PMC3993787

[21] Miorin L, Albornoz A, Baba MM, D’Agaro P, Marcello A. Formation of membrane-defined compartments by tick-borne encephalitis virus contributes to the early delay in interferon signaling. Virus Res. 2012;163(2):660–6. doi: 10.1016/j.virusres.2011.11.020 22155022

[22] Uchida L, Espada-Murao LA, Takamatsu Y, Okamoto K, Hayasaka D, Yu F, et al. The dengue virus conceals double-stranded RNA in the intracellular membrane to escape from an interferon response. Sci Rep. 2014;4:7395. doi: 10.1038/srep07395 25491663 PMC4261170

[23] Neufeldt CJ, Joyce MA, Levin A, Steenbergen RH, Pang D, Shields J, et al. Hepatitis C virus-induced cytoplasmic organelles use the nuclear transport machinery to establish an environment conducive to virus replication. PLoS Pathog. 2013;9(10):e1003744. doi: 10.1371/journal.ppat.1003744 24204278 PMC3814334

[24] Paul D, Bartenschlager R. Flaviviridae replication organelles: oh, what a tangled web we weave. Annu Rev Virol. 2015;2(1):289–310. doi: 10.1146/annurev-virology-100114-055007 26958917

[25] Ci Y, Han K, Kong J, Huang S, Yang Y, Qin C-F, et al. Flavivirus concentrates host ER in main replication compartments to facilitate replication. Adv Sci (Weinh). 2023;10(36):e2305093. doi: 10.1002/advs.202305093 37888856 PMC10754076

[26] Ci Y, Shi L. Compartmentalized replication organelle of flavivirus at the ER and the factors involved. Cell Mol Life Sci. 2021;78(11):4939–54. doi: 10.1007/s00018-021-03834-6 33846827 PMC8041242

[27] Dahmane S, Schexnaydre E, Zhang J, Singh BK, Rosendal E, Chotiwan N, et al. Cryo-electron tomography reveals coupled flavivirus replication, budding and maturation. Nat Commun. 2026;17(1):828. doi: 10.1038/s41467-026-68483-4 41559045 PMC12824359

[28] Abram QH, Landry BN, Wang AB, Kothe RF, Hauch HCH, Sagan SM. The myriad roles of RNA structure in the flavivirus life cycle. RNA Biol. 2024;21(1):14–30. doi: 10.1080/15476286.2024.2357857 38797925 PMC11135854

[29] Mazeaud C, Freppel W, Chatel-Chaix L. The multiples fates of the flavivirus RNA genome during pathogenesis. Front Genet. 2018;9:595. doi: 10.3389/fgene.2018.00595 30564270 PMC6288177

[30] den Boon JA, Nishikiori M, Zhan H, Ahlquist P. Positive-strand RNA virus genome replication organelles: structure, assembly, control. Trends Genet. 2024;40(8):681–93. doi: 10.1016/j.tig.2024.04.003 38724328 PMC13208914

[31] Jones R, Bragagnolo G, Arranz R, Reguera J. Capping pores of alphavirus nsP1 gate membranous viral replication factories. Nature. 2021;589(7843):615–9. doi: 10.1038/s41586-020-3036-8 33328629 PMC7739802

[32] Huang Y, et al. Molecular architecture of coronavirus double-membrane vesicle pore complex. Nature 2024;633:224–31. 39143215 10.1038/s41586-024-07817-y PMC11374677

[33] Wolff G, Limpens RWAL, Zevenhoven-Dobbe JC, Laugks U, Zheng S, de Jong AWM, et al. A molecular pore spans the double membrane of the coronavirus replication organelle. Science. 2020;369(6509):1395–8. doi: 10.1126/science.abd3629 32763915 PMC7665310

[34] Barrows NJ, et al. Biochemistry and molecular biology of flaviviruses. Chem. Rev. 2018;118, 4448–82 (2018). 29652486 10.1021/acs.chemrev.7b00719 PMC5937540

[35] van den Elsen K, Quek JP, Luo D. Molecular insights into the flavivirus replication complex. Viruses. 2021;13(6):956. doi: 10.3390/v13060956 34064113 PMC8224304

[36] Neufeldt CJ, Cortese M, Scaturro P, Cerikan B, Wideman JG, Tabata K, et al. ER-shaping atlastin proteins act as central hubs to promote flavivirus replication and virion assembly. Nat Microbiol. 2019;4(12):2416–29. doi: 10.1038/s41564-019-0586-3 31636417 PMC6881184

[37] Monel B, Rajah MM, Hafirassou ML, Sid Ahmed S, Burlaud-Gaillard J, Zhu P-P, et al. Atlastin endoplasmic reticulum-shaping proteins facilitate zika virus replication. J Virol. 2019;93(23):e01047-19. doi: 10.1128/JVI.01047-19 31534046 PMC6854498

[38] Rismanchi N, Soderblom C, Stadler J, Zhu P-P, Blackstone C. Atlastin GTPases are required for Golgi apparatus and ER morphogenesis. Hum Mol Genet. 2008;17(11):1591–604. doi: 10.1093/hmg/ddn046 18270207 PMC2902292

[39] Hu J, Shibata Y, Zhu P-P, Voss C, Rismanchi N, Prinz WA, et al. A class of dynamin-like GTPases involved in the generation of the tubular ER network. Cell. 2009;138(3):549–61. doi: 10.1016/j.cell.2009.05.025 19665976 PMC2746359

[40] Niu L, Ma T, Yang F, Yan B, Tang X, Yin H, et al. Atlastin-mediated membrane tethering is critical for cargo mobility and exit from the endoplasmic reticulum. Proc Natl Acad Sci U S A. 2019;116(28):14029–38. doi: 10.1073/pnas.1908409116 31239341 PMC6628656

[41] Saini SG, Liu C, Zhang P, Lee TH. Membrane tethering by the atlastin GTPase depends on GTP hydrolysis but not on forming the cross-over configuration. Mol Biol Cell. 2014;25(24):3942–53. doi: 10.1091/mbc.E14-08-1284 25253720 PMC4244202

[42] Orso G, Pendin D, Liu S, Tosetto J, Moss TJ, Faust JE, et al. Homotypic fusion of ER membranes requires the dynamin-like GTPase atlastin. Nature. 2009;460(7258):978–83. doi: 10.1038/nature08280 19633650

[43] Krishna S, Ford MGJ. The atlastin paralogs: the complexity in the tails. J Cell Biol. 2023;222(7):e202305116. doi: 10.1083/jcb.202305116 37327452 PMC10278267

[44] Kinney RM, et al. Construction of infectious cDNA clones for dengue 2 virus: strain 16681 and its attenuated vaccine derivative, strain PDK-53. Virology. 1997;230:300–8. 9143286 10.1006/viro.1997.8500

[45] Münster M, Płaszczyca A, Cortese M, Neufeldt CJ, Goellner S, Long G, et al. A reverse genetics system for Zika virus based on a simple molecular cloning strategy. Viruses. 2018;10(7):368. doi: 10.3390/v10070368 30002313 PMC6071187

[46] Fischl W, Bartenschlager R. High-throughput screening using dengue virus reporter genomes. In: Gong EY, editor. Antiviral methods and protocols. Totowa, NJ: Humana Press; 2013. p. 205–19. 10.1007/978-1-62703-484-5_17 23821271

[47] Russell PK, Udomsakdi S, Halstead SB. Antibody response in dengue and dengue hemorrhagic fever. Jpn J Med Sci Biol. 1967;20 Suppl:103–8. 4968625

[48] Baronti C, Piorkowski G, Charrel RN, Boubis L, Leparc-Goffart I, de Lamballerie X. Complete coding sequence of zika virus from a French polynesia outbreak in 2013. Genome Announc. 2014;2(3):e00500-14. doi: 10.1128/genomeA.00500-14 24903869 PMC4047448

[49] Anton A, Mazeaud C, Freppel W, Gilbert C, Tremblay N, Sow AA, et al. Valosin-containing protein ATPase activity regulates the morphogenesis of Zika virus replication organelles and virus-induced cell death. Cell Microbiol. 2021;23(4):e13302. doi: 10.1111/cmi.13302 33432690

[50] Pizzato M, Erlwein O, Bonsall D, Kaye S, Muir D, McClure MO. A one-step SYBR Green I-based product-enhanced reverse transcriptase assay for the quantitation of retroviruses in cell culture supernatants. J Virol Methods. 2009;156(1–2):1–7. doi: 10.1016/j.jviromet.2008.10.012 19022294

[51] Vermeire J, Naessens E, Vanderstraeten H, Landi A, Iannucci V, Van Nuffel A, et al. Quantification of reverse transcriptase activity by real-time PCR as a fast and accurate method for titration of HIV, lenti- and retroviral vectors. PLoS One. 2012;7(12):e50859. doi: 10.1371/journal.pone.0050859 23227216 PMC3515444

[52] Schindelin J, Arganda-Carreras I, Frise E, Kaynig V, Longair M, Pietzsch T, et al. Fiji: an open-source platform for biological-image analysis. Nat Methods. 2012;9(7):676–82. doi: 10.1038/nmeth.2019 22743772 PMC3855844

[53] Stirling DR, Swain-Bowden MJ, Lucas AM, Carpenter AE, Cimini BA, Goodman A. CellProfiler 4: improvements in speed, utility and usability. BMC Bioinformatics. 2021;22(1):433. doi: 10.1186/s12859-021-04344-9 34507520 PMC8431850

[54] Freppel W, Barragan Torres VA, Uyar O, Anton A, Nouhi Z, Broquière M, et al. Dengue virus and Zika virus alter endoplasmic reticulum-mitochondria contact sites to regulate respiration and apoptosis. iScience. 2024;28(1):111599. doi: 10.1016/j.isci.2024.111599 39834870 PMC11743106

[55] Mastronarde DN. Automated electron microscope tomography using robust prediction of specimen movements. J Struct Biol. 2005;152(1):36–51. doi: 10.1016/j.jsb.2005.07.007 16182563

[56] Posit Team. RStudio: integrated development environment for R. Posit Software PBC; 2025.

[57] R Core Team. R: a language and environment for statistical computing. R Foundation for Statistical Computing; 2022.

[58] Wickham H, François R, Henry L, Müller K, Vaughan D. dplyr: a grammar of data manipulation. 2025.

[59] Wickham H. Ggplot2. Cham: Springer International Publishing. 2016. 10.1007/978-3-319-24277-4

[60] Beygelzimer A, et al. FNN: fast nearest neighbor search algorithms and Applications. 2024.

[61] Kremer JR, Mastronarde DN, McIntosh JR. Computer visualization of three-dimensional image data using IMOD. J Struct Biol. 1996;116(1):71–6. doi: 10.1006/jsbi.1996.0013 8742726

[62] Polishchuk RS, Polishchuk EV, Marra P, Alberti S, Buccione R, Luini A, et al. Correlative light-electron microscopy reveals the tubular-saccular ultrastructure of carriers operating between Golgi apparatus and plasma membrane. J Cell Biol. 2000;148(1):45–58. doi: 10.1083/jcb.148.1.45 10629217 PMC2156208

[63] Polishchuk EV, Polishchuk RS. Pre-embedding labeling for subcellular detection of molecules with electron microscopy. Tissue Cell. 2019;57:103–10. doi: 10.1016/j.tice.2018.11.002 30497685

[64] Goellner S, Cerikan B, Cortese M, Neufeldt CJ, Haselmann U, Bartenschlager R. Replication-independent generation and morphological analysis of flavivirus replication organelles. STAR Protoc. 2020;1(3):100173. doi: 10.1016/j.xpro.2020.100173 33377067 PMC7757399

[65] Cerikan B, Goellner S, Neufeldt CJ, Haselmann U, Mulder K, Chatel-Chaix L, et al. A non-replicative role of the 3’ terminal sequence of the dengue virus genome in membranous replication organelle formation. Cell Rep. 2020;32(1):107859. doi: 10.1016/j.celrep.2020.107859 32640225 PMC7351112

[66] Dobin A, Davis CA, Schlesinger F, Drenkow J, Zaleski C, Jha S, et al. STAR: ultrafast universal RNA-seq aligner. Bioinformatics. 2013;29(1):15–21. doi: 10.1093/bioinformatics/bts635 23104886 PMC3530905

[67] Mudge JM, Carbonell-Sala S, Diekhans M, Martinez JG, Hunt T, Jungreis I, et al. GENCODE 2025: reference gene annotation for human and mouse. Nucleic Acids Res. 2025;53(D1):D966–75. doi: 10.1093/nar/gkae1078 39565199 PMC11701607

[68] Lawrence M, Huber W, Pagès H, Aboyoun P, Carlson M, Gentleman R, et al. Software for computing and annotating genomic ranges. PLoS Comput Biol. 2013;9(8):e1003118. doi: 10.1371/journal.pcbi.1003118 23950696 PMC3738458

[69] Love MI, Huber W, Anders S. Moderated estimation of fold change and dispersion for RNA-seq data with DESeq2. Genome Biol. 2014;15(12):550. doi: 10.1186/s13059-014-0550-8 25516281 PMC4302049

[70] Scaturro P, Stukalov A, Haas DA, Cortese M, Draganova K, Płaszczyca A, et al. An orthogonal proteomic survey uncovers novel Zika virus host factors. Nature. 2018;561(7722):253–7. doi: 10.1038/s41586-018-0484-5 30177828

[71] Tyanova S, Temu T, Cox J. The MaxQuant computational platform for mass spectrometry-based shotgun proteomics. Nat Protoc. 2016;11(12):2301–19. doi: 10.1038/nprot.2016.136 27809316

[72] Tyanova S, Temu T, Sinitcyn P, Carlson A, Hein MY, Geiger T, et al. The Perseus computational platform for comprehensive analysis of (prote)omics data. Nat Methods. 2016;13(9):731–40. doi: 10.1038/nmeth.3901 27348712

[73] Cortese M, Mulder K, Chatel-Chaix L, Scaturro P, Cerikan B, Plaszczyca A, et al. Determinants in nonstructural protein 4A of dengue virus required for RNA replication and replication organelle biogenesis. J Virol. 2021;95(21):e0131021. doi: 10.1128/JVI.01310-21 34379504 PMC8513467

[74] Bowen JR, Quicke KM, Maddur MS, O’Neal JT, McDonald CE, Fedorova NB, et al. Zika virus antagonizes type I interferon responses during infection of human dendritic cells. PLoS Pathog. 2017;13(2):e1006164. doi: 10.1371/journal.ppat.1006164 28152048 PMC5289613

[75] Zimmerman MG, Bowen JR, McDonald CE, Pulendran B, Suthar MS. West Nile Virus infection blocks inflammatory response and T cell costimulatory capacity of human monocyte-derived dendritic cells. J Virol. 2019;93(e00664-19). 10.1128/JVI.00664-19 PMC6854506 31534040

[76] Zimmerman MG, Bowen JR, McDonald CE, Young E, Baric RS, Pulendran B, et al. STAT5: a target of antagonism by neurotropic flaviviruses. J Virol. 2019;93(23):e00665-19. doi: 10.1128/JVI.00665-19 31534033 PMC6854481

[77] Skidmore AM, Bradfute SB. The life cycle of the alphaviruses: from an antiviral perspective. Antiviral Res. 2023;209:105476. doi: 10.1016/j.antiviral.2022.105476 36436722 PMC9840710

[78] Spuul P, Balistreri G, Kääriäinen L, Ahola T. Phosphatidylinositol 3-kinase-, actin-, and microtubule-dependent transport of Semliki Forest virus replication complexes from the plasma membrane to modified lysosomes. J Virol. 2010;84:7543–57. 20484502 10.1128/JVI.00477-10 PMC2897599

[79] Romero-Brey I, Bartenschlager R. Endoplasmic reticulum: the favorite intracellular niche for viral replication and assembly. Viruses. 2016;8(6):160. doi: 10.3390/v8060160 27338443 PMC4926180

[80] Liberzon A, Birger C, Thorvaldsdóttir H, Ghandi M, Mesirov JP, Tamayo P. The Molecular Signatures Database (MSigDB) hallmark gene set collection. Cell Syst. 2015;1(6):417–25. doi: 10.1016/j.cels.2015.12.004 26771021 PMC4707969

[81] Nishikawa M, Nojima S, Akiyama T, Sankawa U, Inoue K. Interaction of digitonin and its analogs with membrane cholesterol. J Biochem. 1984;96(4):1231–9. doi: 10.1093/oxfordjournals.jbchem.a134941 6097588

[82] Fan HY, Heerklotz H. Digitonin does not flip across cholesterol-poor membranes. J Colloid Interface Sci. 2017;504:283–93. doi: 10.1016/j.jcis.2017.05.034 28551523

[83] Prikryl D, Zhang Y, Melikyan GB. Attenuation of IFITM proteins’ antiviral activity through sequestration into intraluminal vesicles of late endosomes. bioRxiv. 2025. doi: 10.1101/2025.05.27.656272 PMC12409171 40918130

[84] Miorin L, Maestre AM, Fernandez-Sesma A, García-Sastre A. Antagonism of type I interferon by flaviviruses. Biochem Biophys Res Commun. 2017;492(4):587–96. doi: 10.1016/j.bbrc.2017.05.146 28576494 PMC5626595

[85] Zoladek J, Nisole S. Mosquito-borne flaviviruses and type I interferon: catch me if you can!. Front Microbiol. 2023;14:1257024. 37965539 10.3389/fmicb.2023.1257024 PMC10642725

[86] Errett JS, Suthar MS, McMillan A, Diamond MS, Gale M Jr. The essential, nonredundant roles of RIG-I and MDA5 in detecting and controlling West Nile virus infection. J Virol. 2013;87(21):11416–25. doi: 10.1128/JVI.01488-13 23966395 PMC3807316

[87] Fredericksen BL, Keller BC, Fornek J, Katze MG, Gale M Jr. Establishment and maintenance of the innate antiviral response to West Nile Virus involves both RIG-I and MDA5 signaling through IPS-1. J Virol. 2008;82(2):609–16. doi: 10.1128/JVI.01305-07 17977974 PMC2224571

[88] Kato H, Takeuchi O, Sato S, Yoneyama M, Yamamoto M, Matsui K, et al. Differential roles of MDA5 and RIG-I helicases in the recognition of RNA viruses. Nature. 2006;441(7089):101–5. doi: 10.1038/nature04734 16625202

[89] Ma J, Ketkar H, Geng T, Lo E, Wang L, Xi J, et al. Zika virus non-structural protein 4A blocks the RLR-MAVS signaling. Front Microbiol. 2018;9:1350. doi: 10.3389/fmicb.2018.01350 29988497 PMC6026624

[90] Sabari S, Chinchankar S, Ambite I, Nazari A, Carneiro APN, Svenningsson A, et al. Rapid ER remodeling induced by a peptide-lipid complex in dying tumor cells. Life Sci Alliance. 2025;8(6):e202403114. doi: 10.26508/lsa.202403114 40132886 PMC11938384

[91] Ricciardi S, Guarino AM, Giaquinto L, Polishchuk EV, Santoro M, Di Tullio G, et al. The role of NSP6 in the biogenesis of the SARS-CoV-2 replication organelle. Nature. 2022;606(7915):761–8. doi: 10.1038/s41586-022-04835-6 35551511 PMC7612910

[92] Crosby D, Mikolaj MR, Nyenhuis SB, Bryce S, Hinshaw JE, Lee TH. Reconstitution of human atlastin fusion activity reveals autoinhibition by the C terminus. J Cell Biol. 2022;221(2):e202107070. doi: 10.1083/jcb.202107070 34817557 PMC8624677

[93] Bian X, Klemm RW, Liu TY, Zhang M, Sun S, Sui X, et al. Structures of the atlastin GTPase provide insight into homotypic fusion of endoplasmic reticulum membranes. Proc Natl Acad Sci U S A. 2011;108(10):3976–81. doi: 10.1073/pnas.1101643108 21368113 PMC3054032

[94] Byrnes LJ, Sondermann H. Structural basis for the nucleotide-dependent dimerization of the large G protein atlastin-1/SPG3A. Proc Natl Acad Sci U S A. 2011;108(6):2216–21. doi: 10.1073/pnas.1012792108 21220294 PMC3038741

[95] Byrnes LJ, Singh A, Szeto K, Benvin NM, O’Donnell JP, Zipfel WR, et al. Structural basis for conformational switching and GTP loading of the large G protein atlastin. EMBO J. 2013;32(3):369–84. doi: 10.1038/emboj.2012.353 23334294 PMC3567502

[96] Liu TY, Bian X, Romano FB, Shemesh T, Rapoport TA, Hu J. Cis and trans interactions between atlastin molecules during membrane fusion. Proc Natl Acad Sci U S A. 2015;112(15):E1851-60. doi: 10.1073/pnas.1504368112 25825753 PMC4403200

[97] Kim KT, Moon Y, Jang Y, Lee KT, Lee C, Jun Y, et al. Molecular mechanisms of atlastin-mediated ER membrane fusion revealed by a FRET-based single-vesicle fusion assay. Sci Rep. 2017;7(1):8700. doi: 10.1038/s41598-017-09162-9 28821793 PMC5562884

[98] Morin-Leisk J, Saini SG, Meng X, Makhov AM, Zhang P, Lee TH. An intramolecular salt bridge drives the soluble domain of GTP-bound atlastin into the postfusion conformation. J Cell Biol. 2011;195(4):605–15. doi: 10.1083/jcb.201105006 22065636 PMC3257528

[99] Winsor J, Machi U, Han Q, Hackney DD, Lee TH. GTP hydrolysis promotes disassembly of the atlastin crossover dimer during ER fusion. J Cell Biol. 2018;217(12):4184–98. doi: 10.1083/jcb.201805039 30249723 PMC6279388

[100] Moss TJ, Andreazza C, Verma A, Daga A, McNew JA. Membrane fusion by the GTPase atlastin requires a conserved C-terminal cytoplasmic tail and dimerization through the middle domain. Proc Natl Acad Sci U S A. 2011;108(27):11133–8. doi: 10.1073/pnas.1105056108 21690399 PMC3131361

[101] Faust JE, Desai T, Verma A, Ulengin I, Sun T-L, Moss TJ, et al. The Atlastin C-terminal tail is an amphipathic helix that perturbs the bilayer structure during endoplasmic reticulum homotypic fusion. J Biol Chem. 2015;290(8):4772–83. doi: 10.1074/jbc.M114.601823 25555915 PMC4335215

[102] Kelly CM, Byrnes LJ, Neela N, Sondermann H, O’Donnell JP. The hypervariable region of atlastin-1 is a site for intrinsic and extrinsic regulation. J Cell Biol. 2021;220:e202104128. 10.1083/jcb.202104128 PMC8563291 34546351

[103] Jang E, Moon Y, Yoon SY, Diaz JAR, Lee M, Ko N, et al. Human atlastins are sufficient to drive the fusion of liposomes with a physiological lipid composition. J Cell Biol. 2023;222(4):e202109090. doi: 10.1083/jcb.202109090 36757370 PMC9949273

[104] Jumper J, Evans R, Pritzel A, Green T, Figurnov M, Ronneberger O, et al. Highly accurate protein structure prediction with AlphaFold. Nature. 2021;596(7873):583–9. doi: 10.1038/s41586-021-03819-2 34265844 PMC8371605

[105] Ota T, Suzuki Y, Nishikawa T, Otsuki T, Sugiyama T, Irie R, et al. Complete sequencing and characterization of 21,243 full-length human cDNAs. Nat Genet. 2004;36(1):40–5. doi: 10.1038/ng1285 14702039

[106] The UniProt Consortium. UniProt: the universal protein knowledgebase in 2025. Nucleic Acids Research. 2025;53:D609–17. 10.1093/nar/gkae1010 PMC11701636 39552041

[107] Green M, Ishino M, Loewenstein PM. Mutational analysis of HIV-1 Tat minimal domain peptides: identification of trans-dominant mutants that suppress HIV-LTR-driven gene expression. Cell. 1989;58(1):215–23. doi: 10.1016/0092-8674(89)90417-0 2752420

[108] García-Nicolás O, Lewandowska M, Ricklin ME, Summerfield A. Monocyte-derived dendritic cells as model to evaluate species tropism of mosquito-borne flaviviruses. Front Cell Infect Microbiol. 2019;9:5. doi: 10.3389/fcimb.2019.00005 30746342 PMC6360178

[109] Schmid MA, Diamond MS, Harris E. Dendritic cells in dengue virus infection: targets of virus replication and mediators of immunity. Front Immunol. 2014;5:647. doi: 10.3389/fimmu.2014.00647 25566258 PMC4269190

[110] Hamel R, Dejarnac O, Wichit S, Ekchariyawat P, Neyret A, Luplertlop N, et al. Biology of Zika virus infection in human skin cells. J Virol. 2015;89(17):8880–96. doi: 10.1128/JVI.00354-15 26085147 PMC4524089

[111] Yin Z, Chen Y-L, Schul W, Wang Q-Y, Gu F, Duraiswamy J, et al. An adenosine nucleoside inhibitor of dengue virus. Proc Natl Acad Sci U S A. 2009;106(48):20435–9. doi: 10.1073/pnas.0907010106 19918064 PMC2787148

[112] Deng Y-Q, Zhang N-N, Li C-F, Tian M, Hao J-N, Xie X-P, et al. Adenosine analog NITD008 is a potent inhibitor of Zika virus. Open Forum Infect Dis. 2016;3(4):ofw175. doi: 10.1093/ofid/ofw175 27747251 PMC5063548

[113] Georgiades P, Allan VJ, Wright GD, Woodman PG, Udommai P, Chung MA, et al. The flexibility and dynamics of the tubules in the endoplasmic reticulum. Sci Rep. 2017;7(1):16474. doi: 10.1038/s41598-017-16570-4 29184084 PMC5705721

[114] Perkins HT, Allan VJ, Waigh TA. Network organisation and the dynamics of tubules in the endoplasmic reticulum. Sci Rep. 2021;11(1):16230. doi: 10.1038/s41598-021-94901-2 34376706 PMC8355327

[115] Barnard TR, Abram QH, Lin QF, Wang AB, Sagan SM. Molecular determinants of flavivirus virion assembly. Trends Biochem Sci. 2021;46(5):378–90. doi: 10.1016/j.tibs.2020.12.007 33423940

[116] Lu C, Xu H, Ranjith-Kumar CT, Brooks MT, Hou TY, Hu F, et al. The structural basis of 5’ triphosphate double-stranded RNA recognition by RIG-I C-terminal domain. Structure. 2010;18(8):1032–43. doi: 10.1016/j.str.2010.05.007 20637642 PMC2919622

[117] Bethell DB, Flobbe K, Cao XT, Day NP, Pham TP, Buurman WA, et al. Pathophysiologic and prognostic role of cytokines in dengue hemorrhagic fever. J Infect Dis. 1998;177(3):778–82. doi: 10.1086/517807 9498463

[118] Chen H-C, Hofman FM, Kung JT, Lin Y-D, Wu-Hsieh BA. Both virus and tumor necrosis factor alpha are critical for endothelium damage in a mouse model of dengue virus-induced hemorrhage. J Virol. 2007;81(11):5518–26. doi: 10.1128/JVI.02575-06 17360740 PMC1900309

[119] Wati S, Li P, Burrell CJ, Carr JM. Dengue virus (DV) replication in monocyte-derived macrophages is not affected by tumor necrosis factor alpha (TNF-alpha), and DV infection induces altered responsiveness to TNF-alpha stimulation. J Virol. 2007;81(18):10161–71. doi: 10.1128/JVI.00313-07 17626094 PMC2045434

[120] Chen Q, Xiao Y, Chai P, Zheng P, Teng J, Chen J. ATL3 is a tubular ER-phagy receptor for GABARAP-mediated selective autophagy. Curr Biol. 2019;29(5):846-855.e6. doi: 10.1016/j.cub.2019.01.041 30773365

[121] Bryce S, Stolzer M, Crosby D, Yang R, Durand D, Lee TH. Human atlastin-3 is a constitutive ER membrane fusion catalyst. J Cell Biol. 2023;222(7):e202211021. doi: 10.1083/jcb.202211021 37102997 PMC10140384

[122] Riva L, et al. The compound SBI-0090799 inhibits Zika virus infection by blocking de novo formation of the membranous replication compartment. J Virol. 2021;95:e0099621. 10.1128/JVI.00996-21 PMC8549497 34468177

[123] Goethals O, Kaptein SJF, Kesteleyn B, Bonfanti J-F, Van Wesenbeeck L, Bardiot D, et al. Blocking NS3-NS4B interaction inhibits dengue virus in non-human primates. Nature. 2023;615(7953):678–86. doi: 10.1038/s41586-023-05790-6 36922586 PMC10033419

[124] Kaptein SJF, Goethals O, Kiemel D, Marchand A, Kesteleyn B, Bonfanti J-F, et al. Publisher Correction: A pan-serotype dengue virus inhibitor targeting the NS3-NS4B interaction. Nature. 2021;599(7883):E2. doi: 10.1038/s41586-021-04123-9 34671169

[125] Wang F, Gao Z, Chen B, Jiang Z, Renner DM, Li J, et al. Modes of action of a small molecule antiviral compound targeting yellow fever virus NS4B protein. Proc Natl Acad Sci U S A. 2025;122(20):e2505498122. doi: 10.1073/pnas.2505498122 40378003 PMC12107144

