# Supplementary material for: Atlastin-2-mediated endoplasmic reticulum membrane tethering is critical for flavivirus replication
Source: PLoS Biol. 2026 Sep 15;24(9):e3003556. doi: 10.1371/journal.pbio.3003556 (PMC13577408; doi:10.1371/journal.pbio.3003556)

Figure 2 Panel E

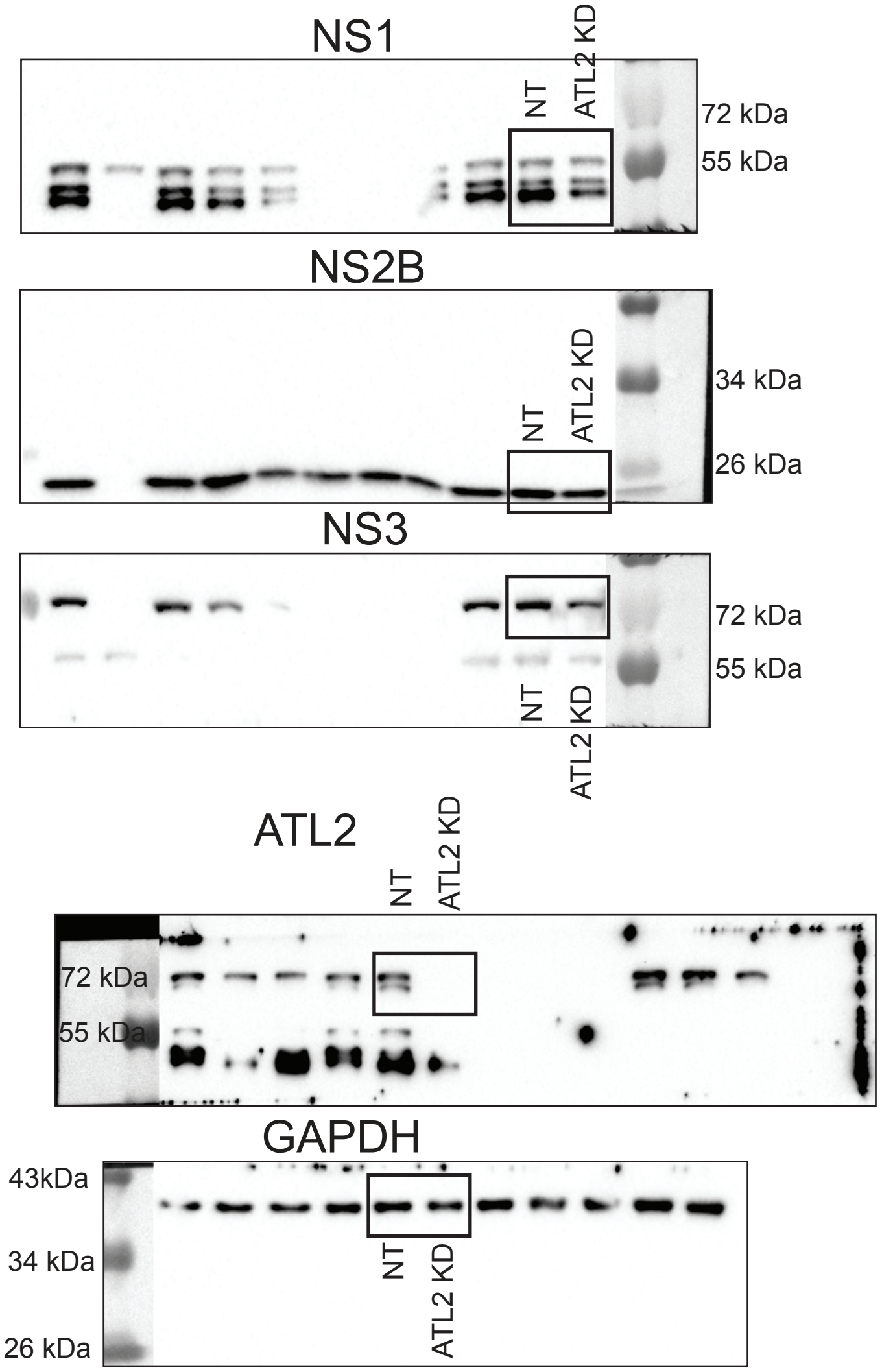

Figure 2 Panel G

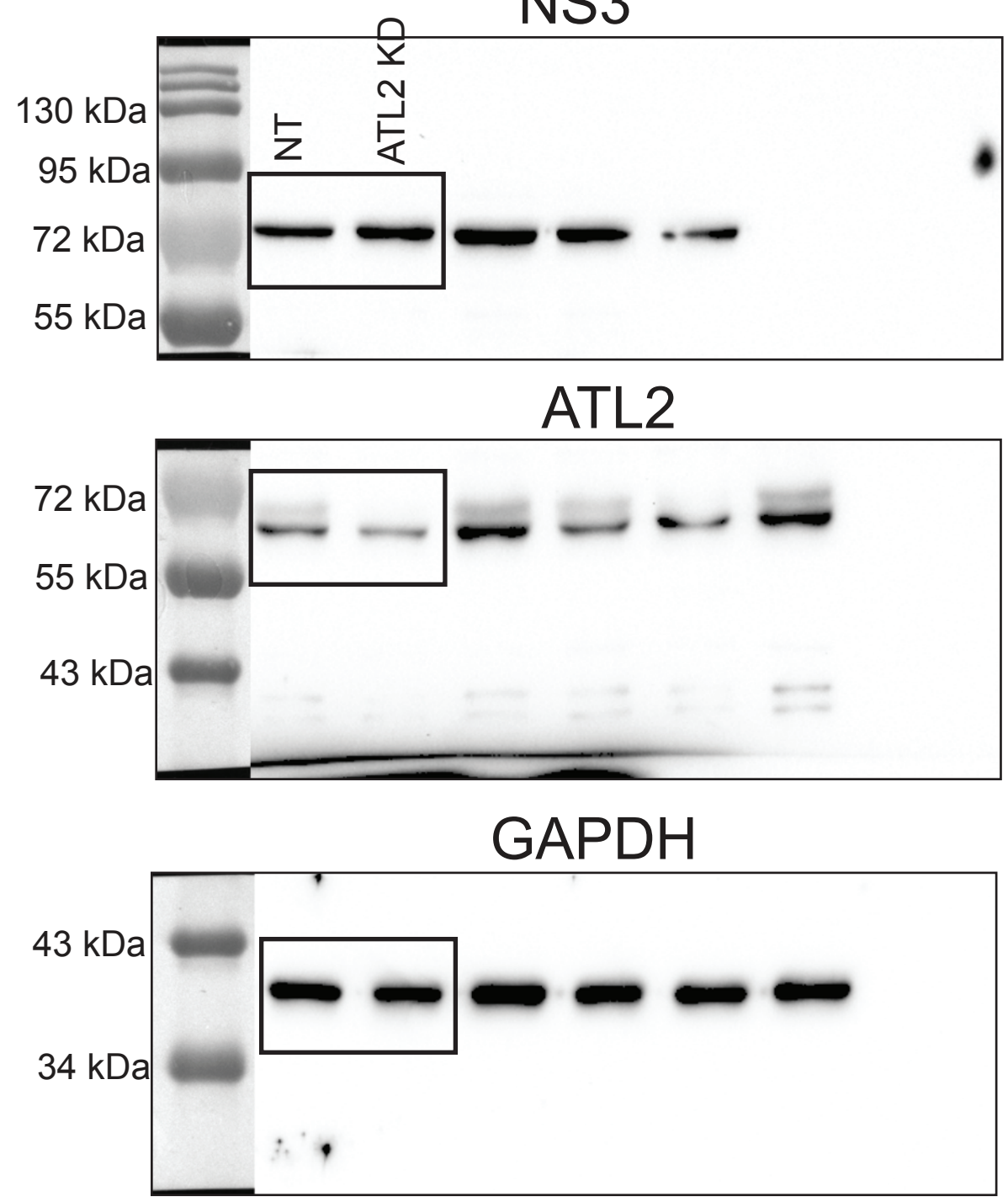

Supp. Figure 1 Panel B

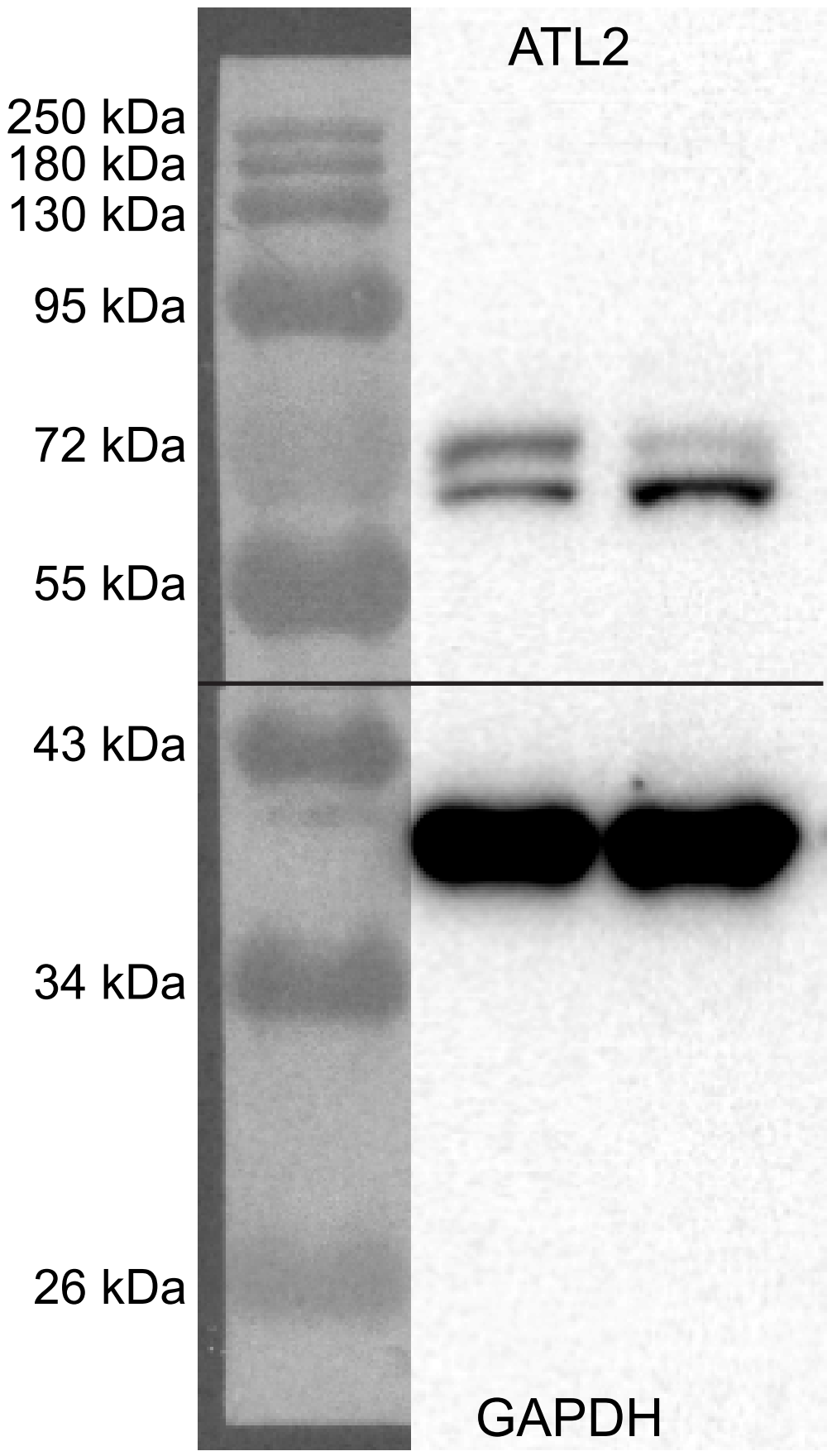

Supp. Figure 5 Panel A

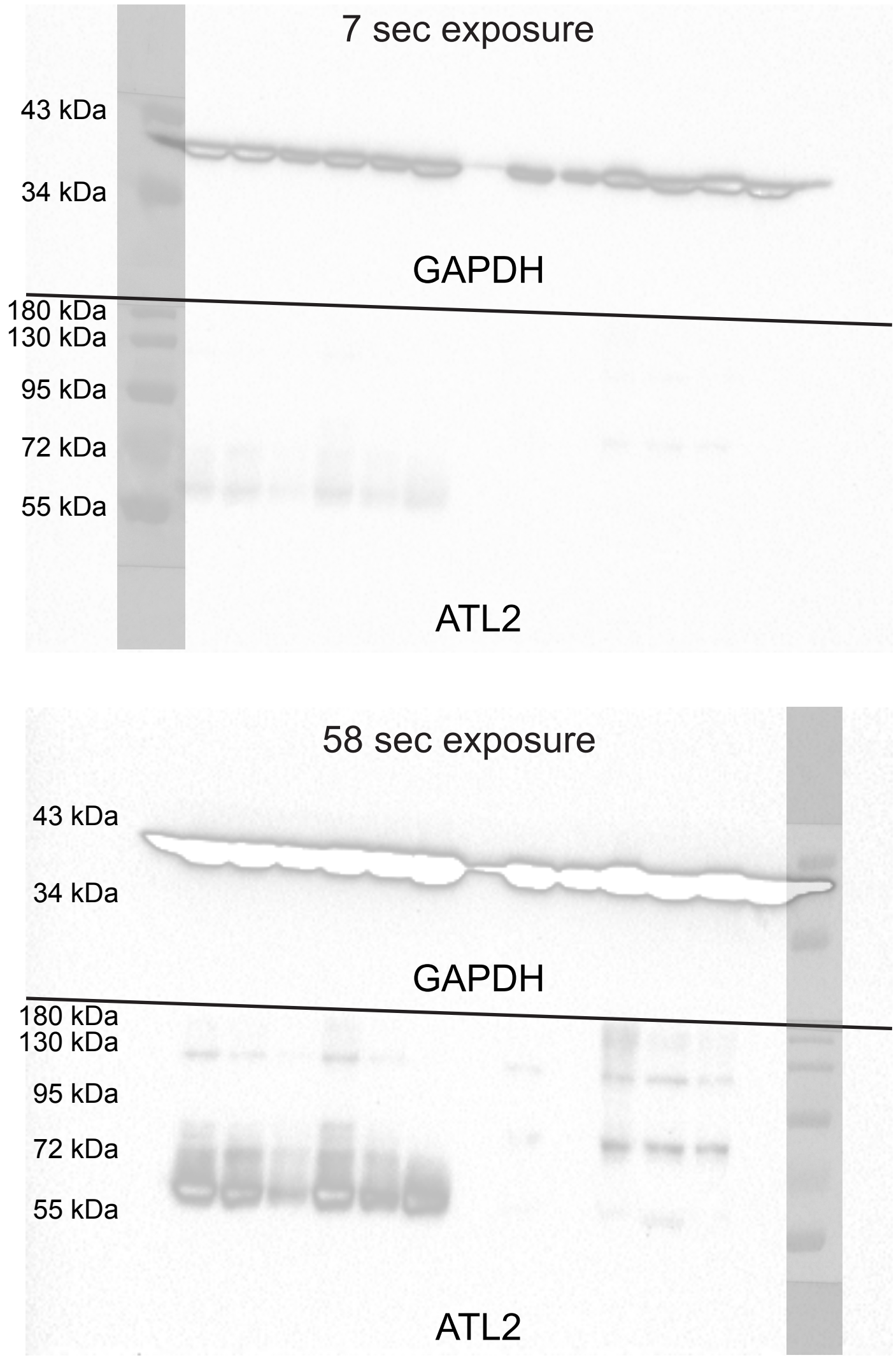

Supp. Figure 5 Panel B

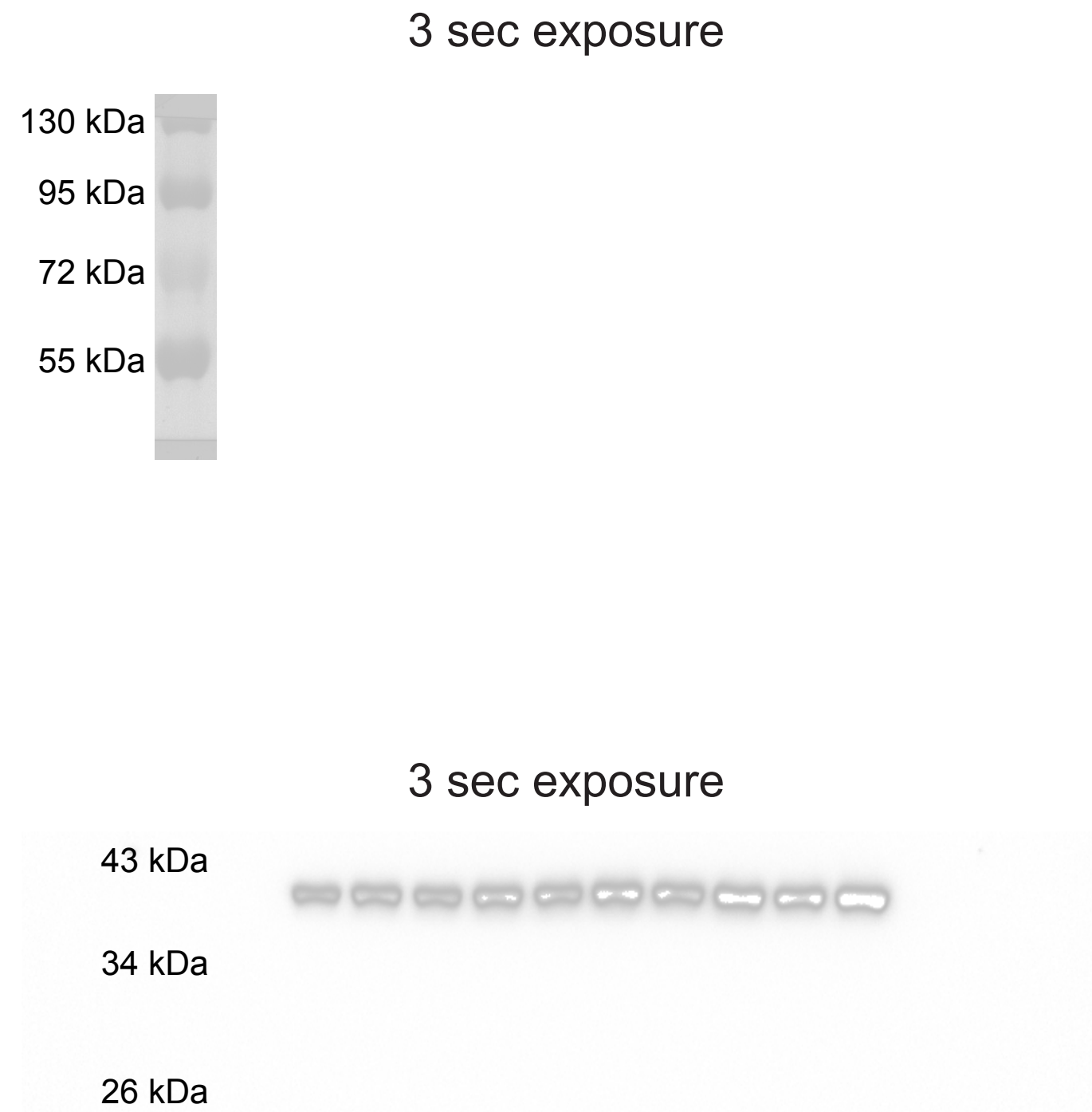

Supplement: Supplementary file 1 [file pbio.3003556.s013.pdf]
