# Supplementary material for: Atlastin-2-mediated endoplasmic reticulum membrane tethering is critical for flavivirus replication
Source: PLoS Biol. 2026 Sep 15;24(9):e3003556. doi: 10.1371/journal.pbio.3003556 (PMC13577408; doi:10.1371/journal.pbio.3003556)
Supplement: Supplementary file 2 [file pbio.3003556.s008.docx]

**S1 Table | Cloning Primers.** List of cloning primers used in this study.

| **Primer** | **Sequence** |
| --- | --- |
| HA-ATL2-fwd | gactagcctcgaggtttatgtacccatacgacgtaccagattacgctgcggagggggacg |
| ATL2-fwd | cgagactagcctcgaggtttatggcggagggggacgag |
| ATL2-1-rvs | tccggcgcgcctgcaggttttcagtctgtctttaatctggcatgatgaga |
| ATL2-2-rvs | tccggcgcgcctgcaggttttctaatttttcttcttgttattgttggatgacagtctctg |
| ATL2-K107A-fwd | ccgtaaaggggcgtcattcctactag |
| ATL2-K107A-rvs | ctagtaggaatgacgcccctttacgg |
| ATL2-P371G-K372E-fwd | acttccacatggagagtccatgcttc |
| ATL2-P371G-K372E-rvs | gaagcatggactctccatgtggaagt |
| ATL2-delta524-583-rvs | tccggcgcgcctgcaggttttcagtatttgacatatgcccaagt |
| HA-ATL2-Q8NHH9-3-fwd | gactagcctcgaggtttatgtacccatacgacgtaccagattacgctgatacccagggtg |
| HA-ATL2-Q8NHH9-4-PCR1-fwd | atgtacccatacgacgtaccagattacgct |
| HA-ATL2-Q8NHH9-4-PCR1-rvs | gaatgacttccctttacggaatgctcctgc |
| HA-ATL2-Q8NHH9-4-PCR2-fwd | tacataatctcaggactgactggcttcattggc |
| HA-ATL2-Q8NHH9-4-PCR2-rvs | ctaatttttcttcttgttattgttggatgacagtctctgtcgc |
| HA-ATL2-Q8NHH9-4-PCR3-fwd | gcattccgtaaagggaagtcattcctactagac |
| HA-ATL2-Q8NHH9-4-PCR3-rvs | gtcagtcctgagattatgtacatagcaaacatgaccg |
| HA-ATL2-Q8NHH9-4-PCR5-fwd | gactagcctcgaggtttatgtacccatacgacgtaccagattacgct |
| HA-ATL2-Q8NHH9-4-PCR5-rvs | tccggcgcgcctgcaggtttctaatttttcttcttgttattgttggatgacagtctc |
| ATL2-C9JQQ5-rvs | tccggcgcgcctgcaggtttttacttaaaatattctacgagatctctacaagtgactttc |
| ATL2-HVD-rvs | tccggcgcgcctgcaggtttttaaagaacaatctgtactggacatggttt |
