# Supplementary material for: Atlastin-2-mediated endoplasmic reticulum membrane tethering is critical for flavivirus replication
Source: PLoS Biol. 2026 Sep 15;24(9):e3003556. doi: 10.1371/journal.pbio.3003556 (PMC13577408; doi:10.1371/journal.pbio.3003556)
Supplement: Supplementary file 3 [file pbio.3003556.s009.docx]

**S2 Table | RT-qPCR primers**. List of RT-qPCR primers used in this study.

| Primer | Sequence |
| --- | --- |
| HPRT_fwd | acaccctttccaaatcctcag |
| HPRT_rvs | cctggcgtcgtgattagtg |
| ATL2_fwd | ttgccacatcctggtcttaaa |
| ATL2_rvs | gcagcaatggaaccagattt |
| DENV_fwd | gcccttctgttcacaccatt |
| DENV_rvs | ccacatttgggcgtaagact |
| ZIKV_fwd | agatgaactgattggccgggc |
| ZIKV_rvs | aggtctcttctgtggaaata |
| WNV_fwd | gggccttctggtcgtgttcttg |
| WNV_rvs | ctggcatgctgatcttggctg |
| POWV_fwd | gagaggttacgcgacactca |
| POWV_rvs | ctctcacggccatgacagat |
| CHIKV_E1_fwd | tttgcgtgccactctgg |
| CHIKV_E1_rvs | cgggtcaccacaaagtacaa |
| ONNV_E1_fwd | tggatagggttcgatacgacacc |
| ONNV_E1_rvs | tcagtacctgctcatcagccca |
| A20_fwd | caggacttgggactttgcga |
| A20_rvs | gtgctctccaacacctctcc |
| IFNb_fwd | actgcaacctttcgaagcct |
| IFNb_rvs | tggaggagacacttgttggtc |
| MX1_fwd | accattccaaggaggtgcag |
| MX1_rvs | tgcgatgtccacttcggaaa |
| OAS1_fwd | cctaccctgtgtgtgtgtcc |
| OAS1_rvs | caaccaggtcagcgtcagat |
| MAVS_fwd | cagcagaaatgaggagaccc |
| MAVS_rvs | gatggcaagatcctcgaagc |
