# Supplementary material for: Atlastin-2-mediated endoplasmic reticulum membrane tethering is critical for flavivirus replication
Source: PLoS Biol. 2026 Sep 15;24(9):e3003556. doi: 10.1371/journal.pbio.3003556 (PMC13577408; doi:10.1371/journal.pbio.3003556)
Supplement: Supplementary file 4 [file pbio.3003556.s010.docx]

**S3 Table | Antibodies.** List of Antibodies used in this study.

| Primary | Supplier | Catalog number |
| --- | --- | --- |
| Anti-dsRNA monoclonal antibody J2 mouse | CedarLane Laboratories | RNT-SCI-10010200 |
| HA-Tag (C29F4) rabbit mAb | Cell Signaling Technology | 3724S |
| Anti-HA antibody, mouse monoclonal | Sigma-Aldrich | H3663 |
| Rat flavivirus NS3 | Anton *et. al.* 2021^49^ | N/A |
| Atlastin-2 polyclonal antibody, rabbit | Bethyl Laboratories | A303-332A |
| Reticulon-3 mouse monoclonal antibody (F-6) | Santa Cruz Biotechnology | sc-374599 |
| Protein disulfide isomerase antibody, rabbit | Sigma-Aldrich | P7496 |
| GAPDH antibody (G-9), mouse | Santa Cruz Biotechnology | sc-365062 |
| Zika virus NS1 antibody, rabbit | GeneTex | GTX133307 |
| Zika virus NS2B protein antibody, rabbit | GeneTex | GTX133308 |
| Zika virus NS5 protein antibody, rabbit | GeneTex | GTX133312 |
| Dengue virus NS5 protein antibody, rabbit | GeneTex | GTX124253 |

| Secondary - IF and immuno-CLEM | Supplier | Catalog number |
| --- | --- | --- |
| Donkey anti-Rabbit IgG, Alexa Fluor 488 | Invitrogen | A21206 |
| Donkey anti-Rabbit IgG, Alexa Fluor 647 | Invitrogen | A31573_41553158 |
| Goat anti-Rat IgG, Alexa Fluor 488 | Invitrogen | A11006 |
| Goat anti-Rat IgG, Alexa Fluor 568 | Invitrogen | A11077_28565870 |
| Goat anti-Rat IgG, Alexa Fluor 647 | Invitrogen | A21247_23996355 |
| Goat anti-Mouse IgG, Alexa Fluor 488 | Invitrogen | A11001 |
| Goat anti-Mouse IgG, Alexa Fluor 568 | Invitrogen | A11004_54244673 |
| Donkey anti-Mouse IgG, Alexa Fluor 594 | Invitrogen | A21203 |
| Donkey anti-Mouse IgG2a, Alexa Fluor 488 | Invitrogen | A21131 |
| Donkey anti-Mouse IgG1, Alexa Fluor 647 | Invitrogen | A21240 |
| STAR ORANGE, goat anti-mouse IgG | abberior | STORANGE-1001-500UG |

| Secondary - Western | Supplier | Catalog number |
| --- | --- | --- |
| Goat Anti-Mouse IgG Peroxidase antibody | MilliporeSigma | A4416-1ML |
| Goat Anti-Rabbit IgG Peroxidase antibody | MilliporeSigma | A6154-1ML |
| Goat Anti-Rat IgG Peroxidase antibody | MilliporeSigma | SAB3700541-2MG |
