# Supplementary material for: Atlastin-2-mediated endoplasmic reticulum membrane tethering is critical for flavivirus replication
Source: PLoS Biol. 2026 Sep 15;24(9):e3003556. doi: 10.1371/journal.pbio.3003556 (PMC13577408; doi:10.1371/journal.pbio.3003556)
Supplement: Supplementary file 5 [file pbio.3003556.s011.docx]

**S4 Table | Peptide Sequences.** Sequences for peptides used in this study.

| **Peptide name** | **Peptide sequence** |
| --- | --- |
| pepTAT-A2 | GRKKRRQRRRPQLKPLGDNLMEENIRQSVTNSIKAGLTDQVSHHARLKTD |
| pepTAT-A2-KKE | GRKKRRQRRRPQLKPLGDNLMKKNIEQSVTNSIKAGLTDQVSHHARLKTD |
| pepTAT | GRKKRRQRRRPQ |
| pepA2 | LKPLGDNLMEENIRQSVTNSIKAGLTDQVSHHARLKTD |
| pepA2-KKE | LKPLGDNLMKKNIEQSVTNSIKAGLTDQVSHHARLKTD |
